# Meta‐analysis of the risk of autoimmune thyroiditis, Guillain‐Barré syndrome, and inflammatory bowel disease following vaccination with AS04‐adjuvanted human papillomavirus 16/18 vaccine

**DOI:** 10.1002/pds.5063

**Published:** 2020-06-24

**Authors:** Dominique Rosillon, Corinne Willame, Fernanda Tavares Da Silva, Adrienne Guignard, Sophie Caterina, Sarah Welby, Frank Struyf

**Affiliations:** ^1^ Research and Development GSK Wavre Belgium

**Keywords:** autoimmune thyroiditis, Guillain‐Barré syndrome, human papillomavirus vaccine, inflammatory bowel disease, pharmacoepidemiology

## Abstract

**Purpose:**

To assess the risk of three autoimmune diseases ‐ autoimmune thyroiditis (AIT), Guillain‐Barré syndrome (GBS), and inflammatory bowel disease (IBD) ‐ in females following AS04‐HPV‐16/18 vaccination.

**Methods:**

This meta‐analysis included data from 18 randomized controlled trials, one cluster‐randomized trial, two large observational retrospective cohort studies, and one case‐control study. Following vaccination, a risk window of 2 years was defined for AIT and IBD and 42 days for GBS. Odds ratios (ORs) were estimated using three methods: meta‐analysis inverse‐variance with continuity correction (primary analysis), pooled estimate, and beta‐binomial regression.

**Results:**

In all studies apart from the case‐control study, 154 398 exposed and 1 504 322 non‐exposed subjects were included, among whom there were 141 and 1972 cases of (autoimmune) thyroiditis; 2 and 2 cases of GBS; and 43 and 401 cases of IBD, respectively. In the case‐control study, there were 97 cases of AIT and 13 of GBS; matched with 802 and 130 controls, respectively. The primary analysis OR estimates were 1.46 (95% confidence interval [CI] 1.22‐1.76), 11.14 (2.00‐61.92), and 1.11 (0.75‐1.66) for (autoimmune) thyroiditis, GBS, and IBD, respectively.

**Conclusions:**

This meta‐analysis did not show an increased risk of IBD following vaccination with AS04‐HPV‐16/18. The 1.5‐fold increased risk of (autoimmune) thyroiditis does not allow us to conclude about a causal association. For GBS, the very low number of cases and wide 95% CIs negate any firm conclusion.

**KEY POINTS**

- There have been hypothesized concerns that vaccine adjuvants could lead to autoimmune conditions.
- This meta‐analysis examined cases of (autoimmune) thyroiditis, Guillain‐Barré syndrome, and inflammatory bowel disease following AS04‐HPV‐16/18 vaccination using all available data from clinical and post‐marketing observational studies.
- This analysis did not show a risk of inflammatory bowel disease.
- There was a 1.5‐fold increased risk of (autoimmune) thyroiditis, but no conclusion about a causal association could be drawn.
- The very low number of cases of Guillain‐Barré syndrome negated any firm conclusion.

## 1 INTRODUCTION

Three human papillomavirus (HPV) vaccines are currently available: AS04‐adjuvanted HPV‐16/18 (AS04‐HPV‐16/18) vaccine (*Cervarix*; GSK)^1^; quadrivalent HPV‐6/11/16/18 (*Gardasil*; Merck Sharp & Dohme Limited),^2^ and a nonavalent HPV vaccine (*Gardasil 9*; Merck Sharp & Dohme Limited).^3^ All three vaccines contain antigens for the high‐risk types HPV‐16 and HPV‐18. AS04‐HPV‐16/18 also contains AS04 ‐ an adjuvant system containing 3‐*O*‐desacyl‐4′‐monophosphoryl lipid A (50 μg MPL) adsorbed on aluminium hydroxide (500 μg Al^3+^)^1^ to boost the immune response.^4^ The other two HPV vaccines contain amorphous aluminium hydroxyphosphate sulphate adjuvant.^2^, ^3^

For many years, there have been alleged concerns that vaccines, per se, may be linked with autoimmune diseases and, more recently, that immunostimulating adjuvants may cause/trigger autoimmune diseases.^5^, ^6^, ^7^, ^8^

During the development of AS04‐HPV‐16/18, clinical trial data^9^, ^10^, ^11^, ^12^, ^13^, ^14^, ^15^, ^16^, ^17^, ^18^, ^19^, ^20^, ^21^, ^22^, ^23^, ^24^, ^25^, ^26^ did not indicate an increased risk of autoimmune diseases. As part of its safety monitoring, two pooled analyses of AS04‐HPV‐16/18 clinical trials were undertaken,^27^, ^28^ examining a wide range of autoimmune events. The second and most comprehensive included 33 339 exposed and 24 241 non‐exposed subjects.^28^ Neither showed an increased risk of autoimmune diseases following AS04‐HPV‐16/18 vaccination.^27^, ^28^ However, two post‐licensure observational studies identified potential safety signals for autoimmune thyroiditis (AIT) and Guillain‐Barré syndrome (GBS) after vaccination with AS04‐HPV‐16/18; and for GBS and inflammatory bowel disease (IBD) following vaccination with HPV‐6/11/16/18.^29^, ^30^

In order to test these signals, we performed a meta‐analysis to estimate the risk of AIT, GBS, and IBD in females following vaccination with AS04‐HPV‐16/18.

## 2 METHODS

### 2.1 Study selection

This meta‐analysis included data from randomized controlled trials (RCTs) and post‐marketing observational studies that were identified in GSK internal repository of studies sponsored and supported by the Company, and information from Regulatory Authorities. In a complementary, systematic literature review searching for all studies published till end 2015. Details of the search strategy, the database consulted and number of references found and selected are described in Data S1. No study additional to those included in the GSK internal repository was found. AS04‐HPV‐16/18 clinical trials had to be interventional RCTs with a non‐HPV vaccine control group in female subjects aged ≥9 years. Extension studies beyond 2 years following first vaccination and ongoing studies on the data lock point date (17 November 2015) were excluded. Post‐marketing observational studies that specifically assessed the association between AS04‐HPV‐16/18 and autoimmune diseases in females were also included.

The following studies were included:

- Eighteen individually RCTs (Data S2)^9^, ^10^, ^11^, ^12^, ^13^, ^14^, ^15^, ^16^, ^17^, ^18^, ^19^, ^20^, ^21^, ^22^, ^23^, ^24^, ^25^, ^26^ that were pooled.
- A cluster‐randomized trial, in which communities of subjects received different vaccination schedules.^31^, ^32^ This study was included separately, due to its large sample size and different safety follow‐up methodology (passive safety surveillance via national registries).
- Two large observational, retrospective cohort studies: a United Kingdom (UK) database cohort study^30^ and a French longitudinal study based on national healthcare administrative databases.^29^, ^33^, ^34^
- A French case‐control study,^35^, ^36^ in which subjects with various autoimmune diseases were matched with controls who met the same general inclusion/exclusion criteria.

### 2.2 Data sources and extraction

Subject‐level data were extracted from all studies except the French cohort study^29^, ^33^, ^34^ and the case‐control study.^35^, ^36^ For these, as individual data were not available, we used aggregated data from publicly available reports. Of note, data from the 2015 report of the French cohort study^29^ were originally used for the AIT analysis (study report available online^37^). However, in 2017, a complementary analysis of the risk of thyroiditis was released^33^ that used a more appropriate methodology for identifying (autoimmune) thyroiditis cases: cases of thyroiditis among those who had previous indicators of thyroiditis were discarded; all cases of thyroiditis reported in both in‐ and out‐patient settings were included; and dates of disease onset were more accurately identified. For AIT, the meta‐analysis includes the results of the French cohort study released in 2017.^33^ However, since the meta‐analysis of AIT including the original results^29^, ^34^ was also performed according to the original statistical analysis plan, we present this analysis for reason of data integrity, in Data S3.

The following data were extracted for each study: numbers of subjects exposed and non‐exposed to AS04‐HPV‐16/18; mean ages; countries of enrollment; length of follow‐up; and numbers of cases of (autoimmune) thyroiditis, GBS, and IBD during the risk period (defined below).

### 2.3 Endpoint case definitions

Clinical definitions of AIT, GBS, and IBD events varied across studies, as detailed in Data S4. Briefly, the clinical studies^9^, ^10^, ^11^, ^12^, ^13^, ^14^, ^15^, ^16^, ^17^, ^18^, ^19^, ^20^, ^21^, ^22^, ^23^, ^24^, ^25^, ^26^ and the cluster‐randomized trial^31^, ^32^ used MedDRA terminology; while the two cohort studies^29^, ^30^, ^33^, ^34^ used International Classification of Diseases, 10th Revision (ICD‐10) codes. The complementary analysis of (autoimmune) thyroiditis in the French cohort study defined cases by the use of thyroid disorder drugs combined with either routine thyroid function tests and complementary examination of the thyroid, or hospital stays with ICD‐10 codes for thyroiditis, or a “new full coverage for thyroiditis as a long‐term illness”.^33^ The UK cohort study^30^ also used “Read codes” classification and only included cases that were confirmed by a medical review of the charts. The case‐control study^35^, ^36^ identified cases of autoimmune disorders through a network of specialist centres at university and general hospitals across France.

In the French cohort study,^29^, ^33^, ^34^ autoimmune and non‐autoimmune thyroiditis cases were included as these were not differentiated in the reports. Therefore, a sensitivity analysis of AIT was performed excluding the French cohort study data.^33^

### 2.4 Risk periods

The post‐vaccination risk periods were determined based on the onset of the disease (acute or insidious) and possible or known pathologic mechanisms.^38^ Irrespective of the underlying mechanisms, it can be assumed that the development of autoimmunity generally requires several weeks ‐ if a causal association between the event and vaccination existed ‐ which is similar to the classical timeframe of several weeks suggested for the onset of post‐infectious autoimmune phenomena.^38^, ^39^

As the clinical courses of AIT and IBD are generally insidious, 2 years between vaccination and disease onset was selected. For trials with longer follow‐up periods, only cases that occurred during the 2 years following first vaccination were included. For the French cohort study,^29^, ^33^, ^34^ only the total numbers of events and the mean follow‐up periods were known. Further, events were reported in exposed subjects and non‐exposed subjects, which included a combination of non‐vaccinated subjects plus the pre‐exposure periods of subjects who were subsequently vaccinated with AS04‐HPV‐16/18 or HPV‐6/11/16/18. Events were therefore estimated as detailed in Data S5.

For GBS, a shorter risk period (42 days following each vaccination) was considered for the main analysis, based on its anticipated acute onset. This period is also recommended by the Brighton Collaboration GBS Working Group,^40^ based on epidemiological data collected after swine flu vaccination during 1976‐1977.^41^, ^42^ For the French cohort study,^29^, ^34^ the time‐to‐onset of the two GBS cases reported among exposed individuals was unknown. We conservatively assumed that these occurred during the 42 days following a vaccination dose and estimated non‐exposed cases as detailed in Data S5. A sensitivity analysis for GBS included cases that occurred during the 2 years following first vaccination.

### 2.5 Statistical methods

To harmonize results across studies, odds ratios (ORs) and 95% confidence intervals (CIs) were calculated from the numbers of cases and total numbers of subjects for the combined clinical studies (Data S2); the cluster‐randomized trial^31^, ^32^; each of the two cohort studies (UK^30^ and French^29^, ^33^, ^34^); and the case‐control study.^35^, ^36^

Meta‐analysis of rare events is challenging due to the inclusion of studies with no event in one or both arms (“single‐zero” and “double‐zero,” respectively).^43^, ^44^ Therefore, three meta‐analysis methods to estimate ORs were used.

In the inverse‐variance method (primary analysis), a continuity correction (please see Data S6) was applied to all studies to overcome the single‐ and double‐zero issue. This method was chosen as the primary analysis because all studies could be included and study heterogeneity could be estimated. In the pooled estimate method, data from all the studies except the case‐control study^35^, ^36^ were pooled and an overall estimate was computed.^45^ The beta‐binomial regression method can include single‐ and double‐zero studies without using continuity correction. Two different beta‐binomial models were analyzed, including, or not, the case‐control study.^35^, ^36^

All statistical analyses were performed using SAS and StatXact‐8.1 procedure for SAS.

## 3 RESULTS

### 3.1 Study population

In 21 studies (all apart from the case‐control study^35^, ^36^), 154 398 exposed (9.3%) and 1 504 322 non‐exposed (90.7%) subjects were included (Table 1). This imbalance was due to the much larger non‐exposed cohort in the French cohort study.^29^, ^34^ The population sizes varied widely between studies, with 19 studies accounting for 3.8% of subjects, and the two cohort studies adding 7.8%^30^ and 88.4%.^29^, ^34^ Exposed subjects were older than non‐exposed subjects (mean age 16.1 vs 13.7 years) due to the imbalance in the French cohort study.^29^, ^34^

**TABLE 1:** Cohort studies included in the meta‐analysis

Study	Number of subjects		Control(s)	Mean age, y		Countries
	Exposed	Non‐exposed		Exposed	Non‐exposed	
Pooled individually randomized clinical trials^9^, ^10^, ^11^, ^12^, ^13^, ^14^, ^15^, ^16^, ^17^, ^18^, ^19^, ^20^, ^21^, ^22^, ^23^, ^24^, ^25^, ^26^ (n = 18)	21 455	20 613	Refer to Data S2	22.1	22.4	Various^a^
Cluster‐randomized trial^b^ ^31^, ^32^	12 400	8119	HBV	14.1	14.1	Finland
UK cohort study^b^ ^30^	64 998	64 994	None	15.3	15.4	UK
French cohort study^c^ ^29^, ^34^	55 545	1 410 596	None	15.0	13.5	France
Total	154 398	1 504 322	‐	16.1	13.7	Various^d^

Abbreviations: HBV, hepatitis B vaccine; UK, United Kingdom.

^a^ Overall: Costa Rica (17.8%), Finland (11.4%), US (10.0%), The Philippines (7.6%), Thailand (5.6%), Brazil (5.5%), Mexico (5.4%), others (<5% each).

^b^ Only females were included.

^c^ Subjects vaccinated with HPV‐6/11/16/18 were excluded. For (autoimmune) thyroiditis, 53 372 exposed and 1 360 003 non‐exposed subjects were considered following re‐analysis.^33^

^d^ Overall: Exposed: UK (42.3%), France (36.0%), Finland (9.6%), others (<5% each); Control: France (93.8%), others (<5% each).

In the aggregated data study (case‐control study),^35^, ^36^ 97 subjects with definite AIT were matched with 802 healthy controls. Only six subjects were exposed to AS04‐HPV‐16/18 vaccine, none of whom developed AIT (ie, all six were in the control group). Thirteen subjects with definite GBS were matched with 130 controls. None of these were vaccinated with AS04‐HPV‐16/18 during the preceding 42 days. IBD was not assessed in this study.

### 3.2 AIT

**FIGURE 1:**
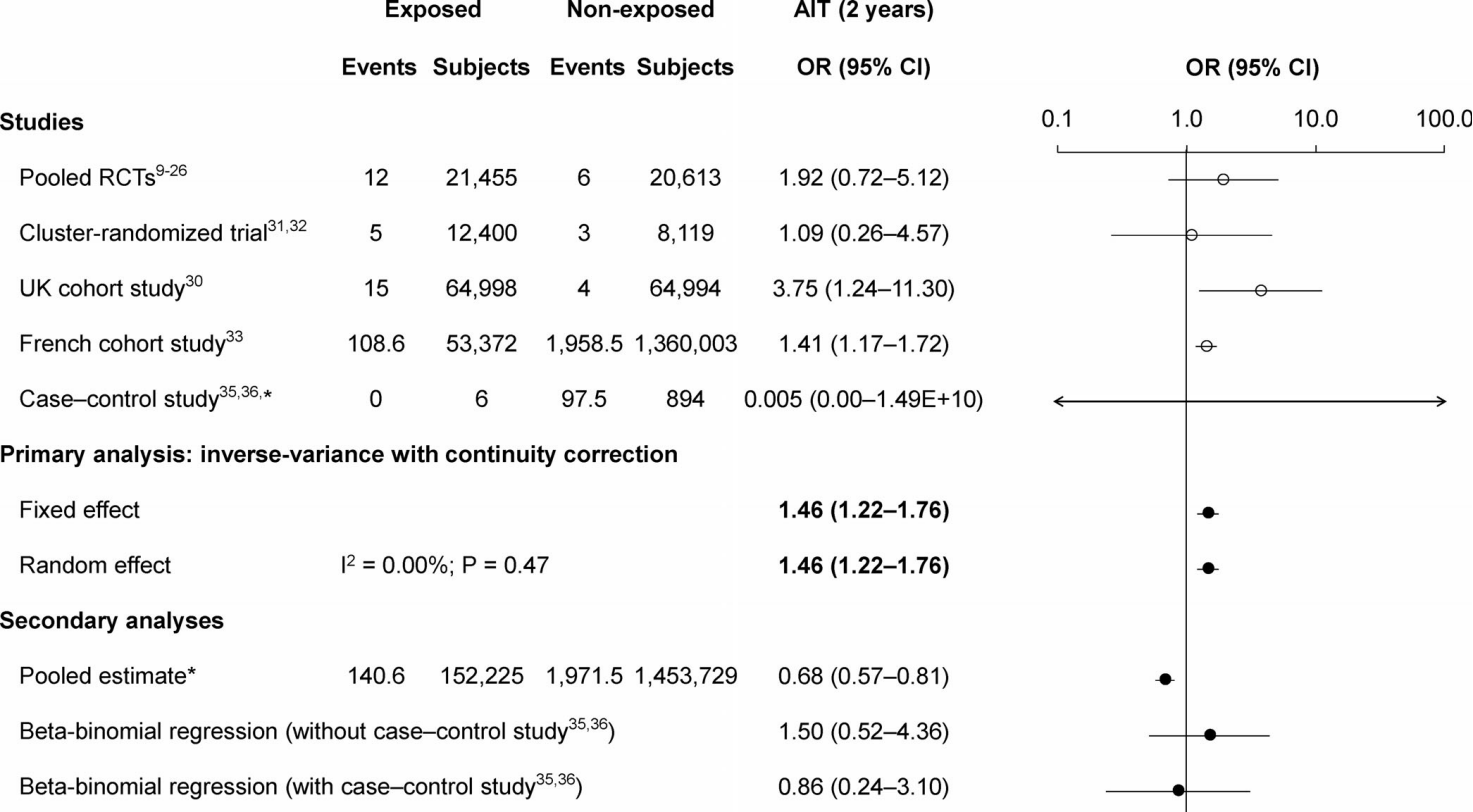
Risk of (autoimmune) thyroiditis during 2 years following the first dose of AS04‐HPV‐16/18. There were partial events for the French cohort study^33^ due to the standardization of the follow‐up time to 2 years; and for the case‐control study^35^, ^36^ due to the continuity correction factor due to the “single‐zero” cases in the exposed arm. AIT, autoimmune thyroiditis; AS04‐HPV‐16/18, AS04‐adjuvanted human papillomavirus‐16/18 vaccine; CI, confidence interval; OR, odds ratio; RCTs, randomized controlled trials; UK, United Kingdom. *The case‐control study was not included in the pooled estimate

There were an estimated 140.6 cases of (autoimmune) thyroiditis among 152 225 exposed subjects (92/100 000) and 1971.5 cases among 1 453 729 non‐exposed subjects (136/100 000). The OR using the inverse‐variance method with continuity correction (primary method) was 1.46 (95% CI 1.22‐1.76), the beta‐binomial regression method without the case‐control study^35^, ^36^ gave a similar OR estimate but had a broader CI, and the pooled OR estimate was 0.68 (95% CI 0.57‐0.81) (Figure 1).

Results using the original analysis of the French cohort study^29^, ^34^ are shown in Data S3 (primary method OR = 2.01 [95% CI 1.30‐3.11]). The sensitivity analysis excluding the French cohort study^33^ provided a primary method OR estimate of 2.15 (95% CI 1.12‐4.14) (Data S7).

### 3.3 GBS

The only GBS cases were from the French cohort study,^29^, ^34^ in which there were two cases of GBS in exposed subjects and 21 cases among non‐exposed subjects (estimated to equate to 1.76 cases during an equivalent follow‐up period in the non‐vaccinated cohort).

**FIGURE 2:**
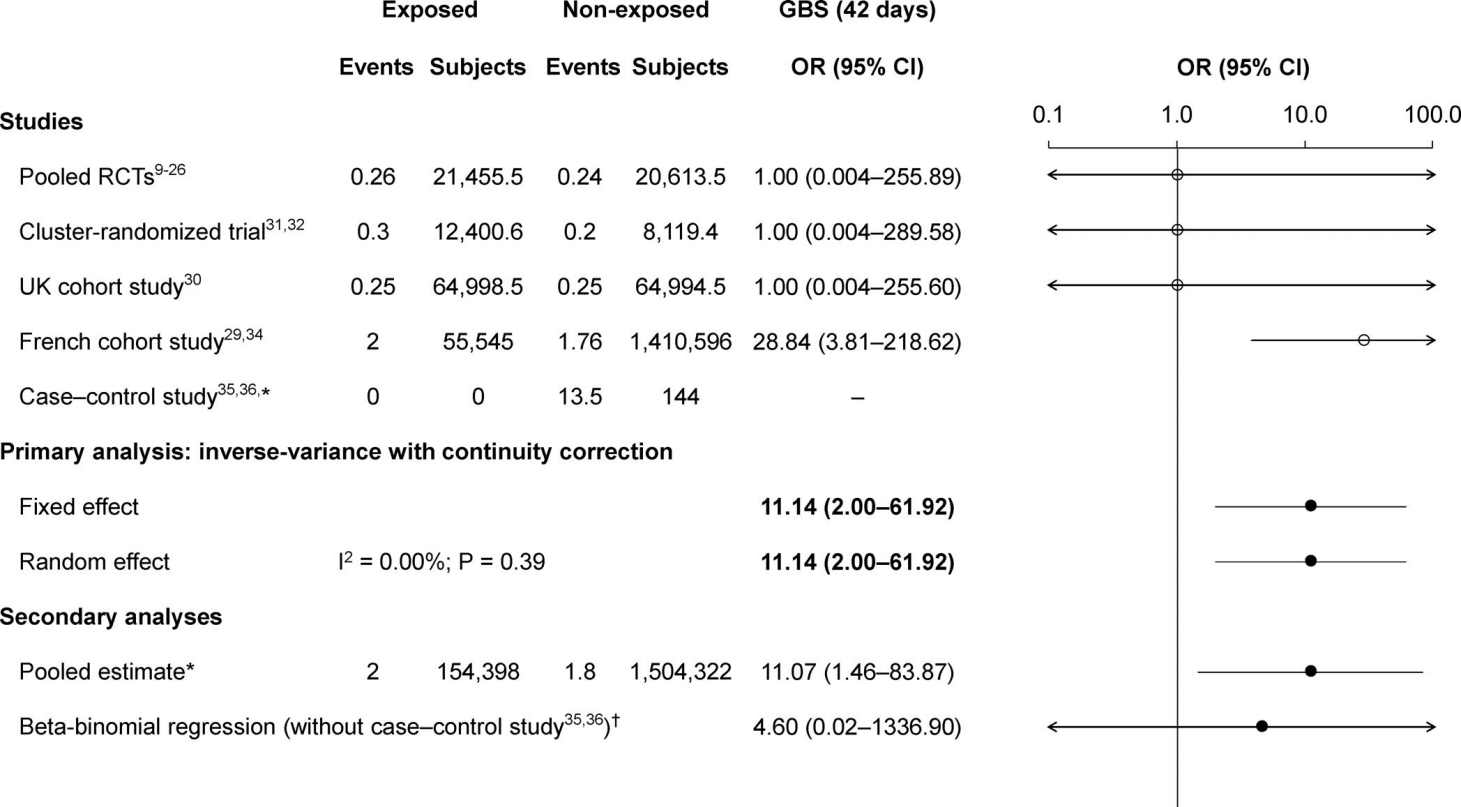
Risk of GBS during 42 days following each dose of AS04‐HPV‐16/18. There are partial events for the non‐exposed arm of the French cohort study^29^, ^34^ due to the standardization of the follow‐up time to 42 days; and for the other studies due to the continuity correction factor due to the “single‐zero” or “double‐zero” cases. AS04‐HPV‐16/18, AS04‐adjuvanted human papillomavirus‐16/18 vaccine; CI, confidence interval; GBS, Guillain‐Barré syndrome; OR, odds ratio; RCTs, randomized controlled trials; UK, United Kingdom.*The case‐control study was not included in the pooled estimate. ^†^The beta‐binomial regression estimate is questionable because of convergence issues

The primary method OR was 11.14 (95% CI 2.00‐61.92; Figure 2). The pooled estimate results were similar, while the beta‐binomial regression method gave a lower estimate, although this was questionable because the low number of cases did not allow model convergence criteria to be met. When the risk period was increased to 2 years, the primary method OR was 3.83 (95% CI 1.08‐13.57), with lower OR estimates using the other methods (Data S8).

### 3.4 IBD

**FIGURE 3:**
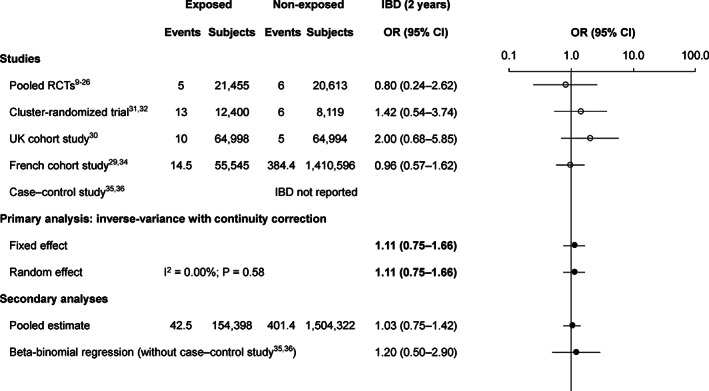
Risk of IBD during 2 years following the first dose of AS04‐HPV‐16/18. There are partial events for the French cohort study^29^, ^34^ due to the standardization of the follow‐up time to 2 years. AS04‐HPV‐16/18, AS04‐adjuvanted human papillomavirus‐16/18 vaccine; CI, confidence interval; IBD, inflammatory bowel disease; OR, odds ratio; RCTs, randomized controlled trials; UK, United Kingdom

There were 42.5 cases of IBD among 154 398 exposed subjects (28/100 000) and 401.4 cases among 1 504 322 non‐exposed subjects (27/100 000). The primary method OR was 1.11 (95% CI 0.75‐1.66); the other methods gave similar estimates (Figure 3).

## 4 DISCUSSION

This meta‐analysis of AS04‐HPV‐16/18 studies was performed to study three autoimmune diseases (AIT, GBS, and IBD) that had been identified as safety signals in observational studies of AS04‐HPV‐16/18 or HPV‐6/11/16/18 vaccines.^29^, ^30^ The analysis included 18 RCTs,^9^, ^10^, ^11^, ^12^, ^13^, ^14^, ^15^, ^16^, ^17^, ^18^, ^19^, ^20^, ^21^, ^22^, ^23^, ^24^, ^25^, ^26^ one cluster‐randomized trial,^31^, ^32^ two large observational, retrospective cohort studies,^29^, ^30^, ^33^, ^34^ and one case‐control study,^35^, ^36^ which combined included approximately 150 000 exposed and 1 500 000 non‐exposed subjects. Risk among females was assessed during pre‐defined risk periods (2 years for AIT and IBD; 42 days for GBS).

The AIT primary analysis showed a slightly increased risk (OR = 1.46) of (autoimmune) thyroiditis following AS04‐HPV‐16/18 vaccination. This is likely a slight overestimation of risk given that this was heavily influenced by the large French cohort study,^33^ for which we calculated an OR of 1.41 (95% CI 1.17‐1.72) based on their crude data, but for which they reported an age‐adjusted hazard ratio (HR) for (autoimmune) thyroiditis of 1.19 (95% CI 0.93‐1.51). Despite this difference, both estimates are similar and <1.5.^46^, ^47^

The other analysis methods showed ambiguous results for AIT due to differences in their weighting of data from different studies. The beta‐binomial model estimate was similar to that from the primary analysis, but the pooled analysis estimate provided an OR of 0.68. This simple pooled estimate is biased because there was a much higher incidence of (autoimmune) thyroiditis in the French cohort study^33^ than in the other studies (exposed: 203 vs 23‐56/100 000; non‐exposed: 144 vs 6‐37/100 000) and the non‐exposed cohort was much larger than the exposed cohort (1 360 003 vs 53 372).^33^ These differences in incidence probably reflect to differences in case definitions (Data S4), as the French cohort study^33^ included all thyroiditis cases (ie, autoimmune and non‐autoimmune), whereas the other studies specifically included AIT, and the French cohort study^33^ included inpatient and outpatient cases. The French cohort study^33^ therefore overcontributed to the incidence in the non‐exposed cohort, resulting in a biased OR < 1. In additional to these limitations, most of the Hill's causal criteria for observational studies such consistency, specificity, coherence, analogy, experimental evidence, etc. were not met.^48^ Similarly, the causality criteria on vaccine adverse events adopted by the Institute of Medicine^49^ were also not encountered: the weight of epidemiological evidence is limited as well as and a plausible biological mechanism has not been identified.^50^ Therefore, there is insufficient evidence to conclude a causal relationship between AS04‐HPV‐16/18 and AIT. Similar findings and conclusions have been published for HPV‐6/11/16/18.^51^

The GBS results were driven by two cases among exposed individuals in the French cohort study,^29^, ^34^ which were conservatively assumed to have occurred within 42 days following vaccination. The French cohort study^29^ reported an adjusted HR of 8.14 (95% CI 1.70‐38.92), while our OR estimate was much higher (28.84 [95% CI 3.91‐218.62]), partly due to the different methodology and lack of age adjustment, but mainly because we conservatively assumed that both GBS cases occurred with 42 days of vaccination. In our sensitivity analysis, when cases of GBS to 2 years were considered, our OR was much more in line with that reported in the French cohort study (3.83 [95% CI 1.08‐13.57]).^29^, ^34^ This was the only study that reported any GBS cases among AS04‐HPV‐16/18‐vaccinated subjects. Given the low number of GBS cases (2/154398 exposed subjects) and the large CI, the risk of GBS following AS04‐HPV‐16/18 vaccination cannot be reliably quantified. Of note, a recent English study found no evidence of an increased risk of GBS during 3 months following vaccination (vs other periods) with AS04‐HPV‐16/18 (relative incidence 0.84; 95% CI 0.30‐2.34).^52^ Further, no increased risk of GBS following vaccination with HPV‐6/11/16/18 has been reported in other studies.^51^, ^53^

The IBD primary analysis did not show an increased risk following AS04‐HPV‐16/18 vaccination. This is in line with previous AS04‐HPV‐16/18 pooled analyses.^27^, ^28^In the current analysis, for every 100 000 subjects vaccinated vs not, IBD was reported for 28 vs 27. This is aligned with results from a 1‐year database cohort study carried out prior to the introduction of HPV vaccines (2005),^54^ in which 35 outpatient cases per 100 000 female adolescents were reported for presumed autoimmune “ulcerative colitis.” These similarities support the lack of an increased risk of IBD with AS04‐HPV‐16/18.

Our results are also supported by a recent systematic review and meta‐analysis that examined the risk of various autoimmune disorders after vaccination with any HPV vaccine.^55^ They reported non‐significant ORs between HPV vaccination and combined autoimmune disorders (1.00 [95% CI 0.95‐1.06]), AIT (1.02 [0.91‐1.14]), GBS (1.28 [0.65‐2.52]), and IBD (1.05 [0.97‐1.14]).^55^ They also examined various other autoimmune disorders and none had a significant association with HPV vaccination apart from a small increased risk of Hashimoto's thyroiditis (1.22 [1.09‐1.36]).^55^

### 4.1 Strengths and limitations

This study highlights the potential value, as well as the limitations of, meta‐analysis as a tool to investigate safety signals related to rare outcomes, which is challenging due to the inclusion of studies with no events in one or both arms.^43^, ^44^ The strength of the meta‐analytical methods employed is that they allowed inclusion of all available information, regardless of the source or study design, resulting in a large sample size. However, as no quality of evidence assessment was performed prior to the meta‐analysis, the only factor contributing to the weight of each study was linked to the size of the population. The results of this meta‐analysis were, therefore, driven by the two largest studies,^29^, ^30^, ^34^ which together contributed 78.1% of exposed and 98.1% of non‐exposed subjects.

There was also heterogeneity between studies, in terms of study design, coding of medical events (Data S4), case ascertainment methods, outcome collection methods, outcome onset identification (eg, diagnosis date vs date of first clinical signs/symptoms), and subject ages. Despite these differences, heterogeneity ‐ as assessed by the *i* ^2^ index (see Figures) ‐ appeared to be very low, although the CIs were very broad, so the apparent lack of heterogeneity should be interpreted with caution.

Regarding study design, the AS04‐HPV‐16/18 exposed and non‐exposed arms of the RCTs should have been balanced by randomization, but there could have been multiple unknown confounders between arms in the observational studies, which it is impossible to adjust for. Also, subjects in both arms could have received additional vaccines, with or without adjuvants, further complicating interpretation of the results. Another difference between the RCTs and observational cohort studies is the level of medical surveillance. In the RCTs, vaccinated and control subjects were followed up according to protocol‐defined scheduled visits. However, in the two observational cohort studies,^29^, ^30^, ^33^, ^34^ cases of autoimmune diseases were diagnosed in routine medical practice. By definition, the risk period in vaccinated subjects started at the time of the first dose, so vaccinated subjects in observational studies would have contact with a healthcare professional for subsequent dose(s), increasing the likelihood that an autoimmune disease would be diagnosed in the exposed vs non‐exposed subjects. Such an “unmasking” effect has been reported in a post‐licensure study of autoimmune diseases following HPV‐6/11/16/18 vaccination.^56^

The case‐control study^35^, ^36^ had a very different design to the other studies, namely it identified girls with autoimmune diseases and matched them with age‐ and place of residence‐matched controls. HPV vaccination status among these two groups was then ascertained. This study could capture events among a large cohort, but the number of subjects included in the study was quite small. It should also be noted that around 1% and 14% of the “non‐exposed” girls in the GBS and AIT analyses, respectively, received HPV‐6/11/16/18.

Case definitions and ascertainment methods varied between studies. In the RCTs, all cases were fully medically validated, whereas in the French cohort study,^29^, ^33^, ^34^ there was no validation, and in the UK cohort study,^30^ there was an intermediate level of validation based on algorithms and patient medical data review. Exposure status validity also varied among studies. Exposure accuracy is close to 100% in clinical trials, but could be uncertain in observational studies. Exposure accuracy was improved in the UK cohort study^30^ by including a historical (pre‐vaccination implementation) non‐exposed cohort. However, in the French cohort study,^29^, ^33^, ^34^ concomitant exposed and non‐exposed cohorts could have reduced exposure accuracy.

Unfortunately, individual subject data (including time‐to‐event data) for the case‐control study^35^, ^36^ and the French cohort study^29^, ^33^, ^34^ were not available, which precluded adjustment for covariates. We had to calculate ORs rather than use their published HRs in order to have a common parameter for all studies. Also in the French cohort study,^29^, ^33^, ^34^ no distinction was made between AIT and non‐autoimmune thyroiditis, limiting the clinical evaluation and interpretation of the findings regarding AIT. For the 42‐day GBS analysis, the number of cases in the French cohort study control group had to be estimated from the overall data, assuming a constant incidence rate. It was assumed that early termination did not depend on exposure and, while this was a reasonable assumption for the other studies (even though subjects could withdraw at any time), this was not the case for the French cohort study. The switch from non‐exposed to exposed status in the French cohort study^29^, ^33^, ^34^ also resulted in a higher mean age for the exposed cohort.

Ideally, disease onset would be from the date of first symptoms, but this is not necessarily known, especially in retrospective database studies. Using diagnosis dates, some autoimmune cases that were diagnosed after vaccination may have had their first symptoms prior to vaccination. Conversely, some cases with first symptoms within 2 years or 42 days following vaccination might have been diagnosed after these windows and therefore not have been included.

Overall, our analysis illustrates that a meta‐analysis can be powerful tool, but its strength is related to the quality of the input data.

## 5 CONCLUSIONS

This meta‐analysis ‐ including approximately 150 000 AS04‐HPV‐16/18‐exposed and 1 500 000 non‐exposed subjects ‐ did not indicate an increased risk of IBD. The results of the analysis showed a 1.5‐fold increased risk of (autoimmune) thyroiditis, but based on existing epidemiological and mechanistic evidence, there is insufficient evidence to conclude a causal association with vaccination. No conclusion regarding the risk of GBS can be drawn as they were driven by two cases among exposed individuals, the times‐to‐onset of which were unknown. Although the GBS OR estimates were high, the number of cases was low and the 95% CIs were wide.

Considering the current results and ongoing surveillance of AS04‐HPV‐16/18 vaccination including other available post‐marketing data and pooled analyses of clinical trial data,^27^, ^28^ there is no evidence to confirm the hypothesis of an association between these autoimmune diseases and AS04‐HPV‐16/18 vaccination.

Given the overall safety data and the demonstrated high and sustained efficacy of AS04‐HPV‐16/18 against HPV‐16/18 infection and cervical lesions,^9^, ^10^, ^11^, ^14^, ^21^, ^57^, ^58^, ^59^ and the potential impact of high‐risk HPV infection (ie, cervical lesions and cervical cancer), we conclude that that the results of the study does not modify the safety and benefit profile of the vaccine.

## ETHICS STATEMENT

The authors state that no ethical approval was needed.

## CONFLICT OF INTEREST

A.G., C.W., D.R., F.T.D.S., S.C., S.W. are employees of the GSK group of companies. A.G., D.R., F.S., F.T.D.S., S.W. hold shares in the GSK group of companies as part of their employee remuneration. F.S. was an employee of the GSK group of companies at the time the study was performed and is currently an employee of Janssen, Pharmaceutical Companies of Johnson & Johnson. F.S. owns shares in the GSK group of companies.

## AUTHOR CONTRIBUTIONS

D.R., C.W. and F.T.D.S. were involved in the design of the study, collection of the data, and interpretation of the results. D.R. and S.C. were involved in the statistical analysis. A.G. and S.C. were involved in the interpretation of the results. F.S. was involved in the design of the study and interpretation of the results.

## Supporting information


**Data S1.** Supporting Information.

**Data S2.** Supporting Information.

**Data S3.** Supporting Information.

**Data S4.** Supporting Information.

**Data S5.** Supporting Information.

**Data S6.** Supporting Information.

**Data S7.** Supporting Information.

**Data S8.** Supporting Information.
