# Supplementary material for: Meta‐analysis of the risk of autoimmune thyroiditis, Guillain‐Barré syndrome, and inflammatory bowel disease following vaccination with AS04‐adjuvanted human papillomavirus 16/18 vaccine
Source: Pharmacoepidemiol Drug Saf. 2020 Jun 24;29(9):1159–67. doi: 10.1002/pds.5063 (PMC7539912; doi:10.1002/pds.5063)
Supplement: Supplementary file 1 — Data S1. Supporting Information. [file PDS-29-1159-s001.zip › PDS_5063_pds-19-0290-File002.docx]

**Supporting information A.** Supplementary systematic literature review – Final Report


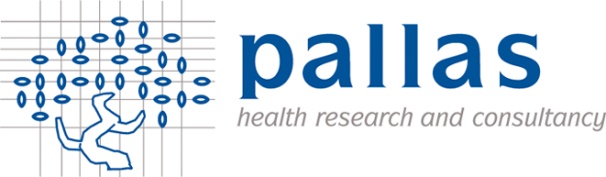


Systematic literature review on Cervarix vaccination and autoimmune diseases

Final report

Rotterdam, January 7th, 2016

E.M. Bunge, PhD

J. Eeuwijk, MSc

F. van Kessel, MSc

Pallas health research and consultancy

www.pallashrc.com


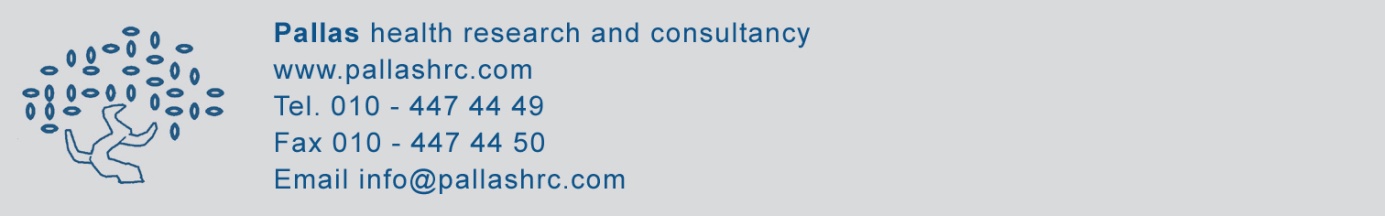


A study commissioned by GlaxoSmithKline Vaccines

**Summary**

*Background*

Cervical cancer is the second leading cause of cancer-related death in women. Oncogenic human papillomavirus (HPV) plays a critical aetiological role in anogenital cancers^1^.

Cervarix® is a bivalent vaccine, containing the virus-like particles (VLPs) of HPV16 and 18, the two types that cause 70% of cervical cancer worldwide, and even greater proportions of HPV-associated vulvar, vaginal, penile, anal, and oropharyngeal cancers^2^. The vaccine was first approved for use in 2007 and is currently licensed in at least 129 countries worldwide, including the US, Canada and European countries^3^.

Recently, GSK has performed a study assessing the risk of autoimmune diseases (AID) in women aged 9-25 years within 1 year after the first dose of Cervarix vaccination (EPI-HPV-040 study). The results of the study did not show any evidence of increased risk for the two co-primary endpoints although an individual disease analysis showed an increased risk of autoimmune thyroiditis. In the meantime, the National French Agency of drugs safety (ANSM-Agence National de Sécurité du Médicament et de produits de santé) provided a report titled ‘HPV vaccines and risk of AID: pharmaco-epidemiological study’. The results of this report show an increased risk of Guillain Barré Syndrome (GBS) and thyroiditis diseases after Cervarix vaccination.

GSK has planned to perform a meta-analysis including if possible the French’s data. The meta-analysis will include GSK clinical trials and GSK pharmaco-epidemiological studies. However, to ensure that the meta-analysis will include any relevant studies, the MAH would like to check if additional studies assessing the risk of AID after Cervarix vaccination are made publicly available

*Objectives*

To identify in the literature potential studies assessing the risk of:

- - Autoimmune thyroiditis
  - Inflammatory bowel diseases (IBD), including ulcerative colitis (UC) and Crohn’s disease (CD)
  - Guillain Barré Syndrome (GBS)

following Cervarix vaccination.

*Methods*

The core of this review was a PubMed and Embase literature search. From the articles retrieved from PubMed and Embase, the relevant references were selected by a three-step selection procedure, based on: 1) screening of title and abstract, 2) screening of full text article, and 3) screening during data-extraction phase. During the second selection step, articles were critically appraised with the SIGN checklist. The search in PubMed and Embase yielded 519 unique hits.

An additional search was performed in Pubmed to check if articles were missed not mentioning autoimmune diseases (AID) in their title or abstract. This search yielded 485 hits.

*Results*

Studies were searched for any report on the occurrence of autoimmune thyroiditis, inflammatory bowel diseases, or Guillain Barré Syndrome in the follow-up period after Cervarix vaccination.

Three studies reported cases of one of these NOAD of interest after vaccination with Cervarix. The follow-up period ranged from twelve months to 36 months. In the first study, all participants received the HPV vaccine in various quantities and/or schedules. Ten subjects reported NOAD, consisting of thyroid disorders and one case each of diabetes mellitus, celiac disease and reactive arthritis. In the second study, no AID was reported in the HPV-16/18 vaccine group while in the control group one case of autoimmune thyroiditis was reported. In the third study, two cases of hypothyroidism were reported in the HPV-vaccine group and one case of hypothyroidism in the control group. All studies were funded or sponsored by GSK.

For the additional search on studies evaluating Cervarix but without mentioning AID in the abstract, no evidence tables were made as all relevant studies were funded or sponsored by GSK.

Eight studies were found in the NIH clinical trial database on bivalent HPV vaccine of which four studies were sponsored by GSK. All studies were still ongoing or recruiting.

*Discussion*

- Many studies reported number of SAE, NOCD or NOAD in general and did not specify AID.
- In some studies, the authors described only vaccine-related AID instead of giving an overview of all AID.
- Many studies were too small to detect rare AID, i.e. autoimmune thyroiditis, inflammatory bowel diseases or Guillain Barré Syndrome.

Contents

[1 Background and objectives 6](#_Toc439846348)

[1.1 Background 6](#_Toc439846349)

[1.2 Research objectives 6](#_Toc439846350)

[1.3 Geographical scope 6](#_Toc439846351)

[2 Methods 7](#_Toc439846352)

[2.1 Analysis international peer reviewed literature 7](#_Toc439846353)

[2.1.1 Literature search 7](#_Toc439846354)

[2.1.2 Additional search 8](#_Toc439846355)

[2.1.3 Selection procedure 9](#_Toc439846356)

[2.1.4 Critical appraisal of the literature 11](#_Toc439846357)

[2.1.5 Data extraction 11](#_Toc439846358)

[2.1.6 Quality control 11](#_Toc439846359)

[2.2 Grey and other literature search 12](#_Toc439846360)

[3 Results 13](#_Toc439846361)

[3.1 Cervarix vaccination and autoimmune disease 13](#_Toc439846362)

[3.2 Grey and other literature search 14](#_Toc439846363)

[4 Discussion 15](#_Toc439846364)

[4.1 Discussion of the results 15](#_Toc439846365)

[4.2 Limitations of the studies 15](#_Toc439846366)

[Abbreviations 16](#_Toc439846367)

[References 17](#_Toc439846368)

[APPENDIX I SIGN Checklists 18](#_Toc439846369)

[APPENDIX II Evidence tables 23](#_Toc439846370)

[APPENDIX III List of studies reporting number of NOAD, but with unclear number of the AID of interest (original search) 27](#_Toc439846371)

[APPENDIX IV List additional search 28](#_Toc439846372)

# Background and objectives

## Background

Cervical cancer is the second leading cause of cancer-related death in women. Oncogenic human papillomavirus (HPV) plays a critical aetiological role in anogenital cancers^1^.

Cervarix® is a bivalent vaccine, containing the virus-like particles (VLPs) of HPV16 and 18, the two types that cause 70% of cervical cancer worldwide, and even greater proportions of HPV-associated vulvar, vaginal, penile, anal, and oropharyngeal cancers^2^. The vaccine was first approved for use in 2007 and is currently licensed in at least 129 countries worldwide, including the United States (US), Canada and European countries^3^.

Recently, GSK has performed a study assessing the risk of autoimmune diseases (AID) in women aged 9-25 years within 1 year after the first dose of Cervarix vaccination (EPI-HPV-040 study). The results of the study did not show any evidence of increased risk for the two co-primary endpoints (neuroinflammatory/ophthalmic diseases and other AID). An individual diseases analysis was performed for the following diseases: autoimmune thyroiditis, Crohn’s disease and Type 1 diabetes mellitus. The results show an increased risk of autoimmune thyroiditis.

The EPI-HPV-040 study was a post-licensure commitment to the US Food and Drug Administration, a non-imposed post-authorisation safety study to the European Medicines Agency and a Post-Authorisation Measure. This study has been submitted to Committee for Medicinal Products for Human Use (CHMP) rapporteur for approval in June 2015.

In the meantime, the National French Agency of drugs safety (ANSM-Agence National de Sécurité du Médicament et de produits de santé) provided a report titled ‘HPV vaccines and risk of AID: pharmaco-epidemiological study’. The results of this report show an increased risk of Guillain Barré Syndrome (GBS) and thyroiditis diseases after Cervarix vaccination.

The CHMP has requested to marketing authorisation holder (MAH) to explore the data of the French study (ANSM report). In the meantime, GSK has committed to CHMP to perform a meta-analysis including if possible the French’s data. The meta-analysis will include GSK clinical trials and GSK pharmaco-epidemiological studies.

However, to ensure that the meta-analysis will include any relevant studies, the MAH would like to check if additional studies assessing the risk of AID after Cervarix vaccination are made publicly available

## Research objectives

To identify in the literature potential studies assessing the risk of:

- - Autoimmune thyroiditis
  - Inflammatory bowel diseases (IBD), including ulcerative colitis (UC) and Crohn’s disease (CD)
  - Guillain Barré Syndrome

following Cervarix vaccination.

## Geographical scope

The geographical scope was worldwide.

# Methods

In order to meet the objectives as outlined in section 1.2, Pallas performed a systematic review of the literature. A systematic review is a method to collect, critically appraise, and summarize the best available evidence in a transparent and systematic way using generally accepted evidence-based principles.

Pallas finalized the search strategies, selected literature based on title and abstracts, critically appraised full-text articles based on checklists for Evidence Based Medicine, and summarized the evidence in consultation with GSK Vaccines. Results were documented in evidence tables and exclusion tables in order to ensure transparency and reproducibility of the results. The review steps are further outlined below.

## Analysis international peer reviewed literature

The core of this review was a PubMed literature search, complemented with a search Embase.

### Literature search

**PubMed**

In order to find relevant articles for the objective, Pallas made a search string consisting of:

1. Terms for disease
   1. Autoimmune thyroiditis
   2. Inflammatory bowel diseases, including ulcerative colitis and Crohn’s disease
   3. Guillain Barré Syndrome
   4. Autoimmune disease in general
2. Terms for Cervarix vaccination

#1A: Autoimmune thyroiditis

"Thyroiditis, Autoimmune"[Mesh] OR autoimmune thyroiditis[tiab] OR auto-immune thyroiditis[tiab] OR "Hashimoto Disease"[Mesh] OR hashimot*[tiab] OR "struma lymphomatosa"[tiab] OR Autoimmune Thyroiditides[tiab] OR Lymphocytic Thyroiditis[tiab] OR Lymphomatous Thyroiditis[tiab] OR Lymphocytic Thyroiditides[tiab] OR Lymphomatous Thyroiditides[tiab]

#1B: Inflammatory bowel disease

“Crohn Disease"[Mesh] OR Crohn*[tiab] OR "Colitis,Ulcerative"[Mesh] OR ulcerative colitis[tiab] OR “Inflammatory bowel diseases”[Mesh] OR Inflammatory Bowel Disease[tiab] OR Colitis Gravis[tiab] OR Idiopathic Proctocolitis[tiab]

#1C: Guillain Barré Syndrome

"Guillain-Barre Syndrome"[Mesh] OR Guillain-Barre Syndrome*[tiab] OR "Miller Fisher Syndrome"[Mesh] OR miller fisher syndrome*[tiab] OR Acute Inflammatory Polyneuropathy[tiab] OR Guillain-Barré Syndrome[tiab] OR Acute Inflammatory Polyradiculoneuropathy[tiab] OR Landry-Guillain-Barre Syndrome[tiab] OR Landry Guillain Barre Syndrome[tiab] OR Acute Autoimmune Neuropathy[tiab] OR Acute Infectious Polyneuritis[tiab] OR Ophthalmoplegia, Ataxia and Areflexia Syndrome[tiab]

#1D: Autoimmune disease (general terms)

"Autoimmune Diseases"[Mesh] OR autoimmune[tiab] OR auto-immune[tiab]

#2. Cervarix vaccination

Cervarix[tw] OR “human papillomavirus vaccine, L1 type 16, 18”[Supplementary Concept] OR HPV L1 vaccine, bivalent 16,18[tiab] OR HPV-16/18 vaccine[tiab] OR human papillomavirus vaccine L1 16,18[tiab] OR HPV-16/18 AS04-adjuvanted vaccine[tiab] OR “Papillomavirus Vaccines”[Mesh] OR Human Papillomavirus Vaccine*[tiab] OR HPV Vaccine*[tiab]

Limits

No limits were applied.

Number of hits

The combination of these search strings, i.e. #1 (1A OR 1B OR 1C OR 1D) AND #2, yielded 112 hits (dd. November 18th 2015).

**Embase**

The Embase search was based on the same search strings as the PubMed search.

#1A: Autoimmune thyroiditis

'autoimmune thyroiditis'/exp OR (‘autoimmune thyroiditis’ OR ‘auto-immune thyroiditis’):ti,ab OR 'Hashimoto disease'/exp OR (hashimot* OR ‘struma lymphomatosa’ OR ‘Autoimmune Thyroiditides’ OR ‘Lymphocytic Thyroiditis’ OR ‘Lymphomatous Thyroiditis’ OR ‘Lymphocytic Thyroiditides’ OR ‘Lymphomatous Thyroiditides’):ti,ab

#1B: Inflammatory bowel disease

'Crohn disease'/exp OR Crohn*:ti,ab OR 'ulcerative colitis'/exp OR ‘ulcerative colitis’:ti,ab OR 'inflammatory bowel disease'/exp OR (‘Inflammatory Bowel Disease’ OR ‘Colitis Gravis’ OR ‘Idiopathic Proctocolitis’):ti,ab

#1C: Guillain Barré Syndrome

'Guillain Barre syndrome'/exp OR (‘Guillain-Barre Syndrome’ OR ‘Guillain-Barré Syndrome’ OR ‘miller fisher syndrome’ OR ‘Acute Inflammatory Polyneuropathy’ OR ‘Acute Inflammatory Polyradiculoneuropathy’ OR ‘Landry Guillain Barre Syndrome’ OR ‘Acute Autoimmune Neuropathy’ OR ‘Acute Infectious Polyneuritis’ OR ‘Ophthalmoplegia, Ataxia and Areflexia Syndrome’):ti,ab

#1D: Autoimmune disease (general terms)

'autoimmune disease'/exp OR (autoimmune OR auto-immune):ti,ab

#2 Influenza

‘Cervarix’/exp OR (‘human papillomavirus vaccine L1 type 16 18’ OR ‘HPV-16/18 vaccine’ OR 'hpv-16/18 vaccine' OR ‘HPV-16/18 AS04-adjuvanted vaccine’):ti,ab OR 'Wart virus vaccine'/exp OR (‘Human Papillomavirus Vaccine’ OR 'human papillomavirus vaccines'):ti,ab OR (hpv AND vaccine*:ti,ab)

Limits

No limits were applied.

Number of hits

The combination of these search strings, i.e. #1 (1A OR 1B OR 1C OR 1D) AND #2, yielded 488 hits (dd. November 18th 2015).

### Additional search

An additional search was performed to check if articles were missed not mentioning autoimmune diseases in their title or abstract. References already included in the original search or hand search were excluded from the additional search.

**PubMed**

*Cervarix vaccination*Cervarix[tw] OR “human papillomavirus vaccine, L1 type 16, 18”[Supplementary Concept] OR HPV L1 vaccine, bivalent 16,18[tiab] OR HPV-16/18 vaccine[tiab] OR human papillomavirus vaccine L1 16,18[tiab] OR HPV-16/18 AS04-adjuvanted vaccine[tiab] OR hpv bivalent vaccine[tiab]) OR 16 18 as04[tiab]Limits

Limits

No limits were applied.

Number of hits

The search yielded 485 hits (dd. December 3^rd^ 2015).

### Selection procedure

From the articles retrieved from PubMed and Embase, the relevant references were selected by a three-step selection procedure, based on:

1. Screening of title and abstract (first selection step): In this step, articles that seemed to contain relevant data for the objectives based on the title and abstract were selected for full-text screening, while articles that did not seem to contain relevant data were not selected. In case of doubt, the article was checked full-text in the second selection step.

- Inclusion criteria:
- Data relevant for the objectives.
- Reasons for exclusion were:
- Studies in boys;
- Studies on Auto-inflammatory Syndrome induced by Adjuvants (ASIA);
- Populations pre-existing AID;
- Studies on Gardasil.

1. Screening of full article (second selection step): In this step the full text of the articles selected in step 1 were assessed. First it was determined whether the article answered one of the review questions. If this was the case, then the article was critically appraised using a standard set of criteria (see section 2.1.5). Reasons for exclusion in this stage were:

- A narrative review (e.g. no methods section that described the way the authors collected the literature);
- Non-pertinent publication type
- Gardasil or non-Cervarix 2vHPV vaccine (i.e. vaccine produced by a Chinese Pharmaceutical company);
- Phase I or phase II studies with small sample size and short follow-up period;
- Insufficient methodological quality;
- No quantitative data could be retrieved from the article.

1. Screening during data-extraction phase (third selection step): further scrutiny of the article during the data-extraction phase might lead to exclusion:

- During the screening of full articles systematic reviews and meta-analysis were selected. The reference lists of these articles were checked on possibly missed relevant articles and thereafter excluded.
- Systemic reviews and meta-analysis that were checked were:
  - Agorastos, T., et al., 2009
  - Angelo, M.G., et al., 2014
  - De Vincenzo, R.C., et al., 2014
  - Macartney, K.K., et al., 2013
  - Medeiros, L.R., et al., 2009
  - Pellegrino. P., et al., 2014
  - Stillo, M.P., et al., 2015

Recording of process

The process of selection and inclusion and exclusion of articles was registered in an Endnote library by one of the researchers. In this way, a clear overview of all selection steps was maintained at all phases and this assured reproducibility of the results.

Figure 1a gives a schematic overview of the selection procedure for the original objectives, including the number of articles found and retrieved from PubMed and Embase and the final number of articles included. Figure 1b gives a schematic overview of the selection procedure performed on the results of the additional search in PubMed (see 2.1.2).

**Figure 1a. Selection procedure PubMed and Embase**

PubMed search

N=112

Embase search

n=488

Unique hits

n=519

Selected based on title and abstract

n=53

Full text selection

n=45

Included in evidence tables

n=3

Excluded based on title and abstracts

n=466

Not available in full text

n=8

Excluded: n=43

- Narrative review (n=12)
- Non-pertinent publication type (n=11)
- Systematic review (n=7)
- Pooled analysis (n=3)
- Study does not answer study objectives (prevaccination period and estimated cases) (n=2)
- Study on Gardasil (n=1)
- AID in general (n=7)

Included from hand search

n=1

**Figure 1b. Selection procedure additional search in PubMed**

PubMed search

n=485

Selected based on title and abstract

n=61

Full text selection

n=52

Included in evidence tables

n=0

Excluded based on title and abstracts

n=424

Not available in full text

n=9

Excluded: n=52

- Nothing reported on adverse events (AE) (n=10)
- Narrative review (n=3)
- Phase 1 study (n=2)
- Follow-up period too short, e.g. seven days (n=3)
- No Cervarix vaccination (n=1)
- Booster dose after qHPV of bHPV after qHPV vaccination (n=1)
- AE, but no cases of AID (n=13)
- AE, but not clear if AID were present (n=9)
- NOAD in general (n=10)

### Critical appraisal of the literature

The Pallas team critically appraised the methodological quality of the articles using the SIGN checklist (appendix I).

### Data extraction

Relevant articles on the occurrence of autoimmune thyroiditis, IBD, or GBS in the follow-up period after Cervarix vaccination identified during the literature search in PubMed and Embase (see 2.1.1) were summarised using standardised data-extraction tables (evidence tables) in Word (appendix II).

In addition, a list is made of studies reporting the number of serious adverse events (SAE), new-onset chronic disease (NOCD) or new-onset autoimmune disease (NOAD), but without mentioning the exact number of the AIDs of interest in the treatment arms(appendix III). These articles are not summarized in evidence tables.

As agreed with GSK, for the additional search, no evidence tables were made as all relevant studies were funded or sponsored by GSK. Three lists were made (appendix IV):

- A list of studies presenting the number of SAE during the follow-up period , including the number of AID;
- A list of studies presenting the number of SAE during the follow-up period, but without further specifying which disease were found;
- A list of studies presenting the number of NOAD, but without further specifying the type of AID.

### Quality control

The following quality control measures were put in place:

- The first 30% of titles and abstracts were screened in duplicate by two independent researchers from Pallas. The results were compared and discussed before the remaining references were assessed by one researcher. The differences between the two researchers were less than 5% with regard to the articles screened in duplicate.
- The first 10% of full text articles were critically appraised in duplicate by two independent researchers from Pallas. The results were compared and discussed early in the process. Any disagreements were adjudicated by a third researcher when necessary. The differences between the two researchers were less than 5% with regard to the articles screened in duplicate.
- Data extraction: the evidence tables were compiled by junior researchers and reviewed by the senior researcher of the project.

## Grey and other literature search

The focus of this literature review was on peer-reviewed articles with sufficient quality, hence no grey literature search was performed. The National Institutes of Health (NIH) website ‘www.clinicaltrials.gov’ was checked (dd. January 4^th^ 2016) for ongoing trials on Cervarix vaccination. In total, 243 trials were found in the database with a search for ‘cervical cancer vaccine’. Studies with an unknown status were excluded.

In addition, the report entitled ‘Vaccins anti-HPV et risque de maladies autoimmunes: étude pharmacoépidémiology by the Agence Nationale de sécurité du Médicament et des produits de santé’ published in September 2015, was checked for relevant references.

# Results

In total three articles were included in this review. In all three, thyroid disease was reported after Cervarix vaccination as SAE ^4-6^. Inflammatory bowel disease or Guillain Barré Syndrome were not reported.

The results will be summarized in this chapter. More detailed results are presented in the evidence tables in Appendix II.

## Cervarix vaccination and autoimmune disease

In three studies, new-onset autoimmune diseases were reported after vaccination with Cervarix. Two studies were multi-country studies^4 5^. One study was performed in Brazil ^6^. The follow-up period ranged from twelve months to 36 months.

In the study of Romanowski et al., healthy girls and young women aged 9 to 25 years were randomized to one of the four HPV-vaccination groups. Group 1 received three doses of HPV-16/18 (20 μg/20 μg) vaccine at months 0, 1 and 6, group 2 received two doses of HPV-16/18 (20 μg/20 μg) vaccine at months 0 and 6, group 3 received two doses of HPV-16/18 (40 μg/40 μg) vaccine at months 0 and 6 and group 4 received two doses of HPV-16/18 (40 μg/40 μg) vaccine at months 0 and 2. In both group 1 and 2, 1.3% (three cases) reported NOAD while in both group 3 and 4, 0.8% (two cases) reported NOAD. NOADs consisted of thyroid disorders and one case each of diabetes mellitus, celiac disease and reactive arthritis. It was not stated in which groups the thyroid disorders were reported^5^.

In another study, conducted in 57 centres in twelve different countries, healthy girls aged 10–14 years were randomized to receive HPV-16/18 vaccine in a 0-, 1-, 6-month schedule or a hepatitis A vaccine . One SAE in the control group was autoimmune thyroiditis. No autoimmune-diseases were reported in the HPV-16/18 vaccine group^4^.

In the study of Naud et al., healthy women aged 15-25 years were recruited and randomized to receive HPV-16/18 vaccine or placebo. In the HPV-vaccine group, 0.9% (2 cases) reported hypothyroidism between 77 months to 114 months post initial vaccination while 0.5% (1 case) from the placebo group reported hypothyroidism between 77 months to 114 months post initial vaccination ^6^.

Table 1. Included studies with reported new-onset autoimmune disease

| **Ref**  **Trialnr** | **Country** | **Vaccine schedule** | **n** | **Follow-up** | **Outcome** |
| --- | --- | --- | --- | --- | --- |
| Romanowski, 2011  NCT00541970 | Canada, Germany | 1) 3-dose 20/20 M0,1,6  2) 2-dose 20/20 M0,6  3) 2-dose 40/40 M0,6  4) 2-dose 40/40 M0,2 | 960  1) n=239  2) n=240  3) n=241  4) n=240 | 24 months | Subjects with NOAD, % (n)  1) 1.3% (3)  2) 1.3% (3)  3) 0.8% (2)  4) 0.8% (2)  Reported NOAD consisted of thyroid disorders and one case each of diabetes mellitus, celiac disease and reactive arthritis |
| Rivera Medina, 2010  NCT00196924 | Australia, Colombia, the Czech Republic, France, Germany, Honduras, Korea, Norway, Panama, Spain, Sweden, Taiwan | 1) HPV-16/18 vaccine: 20mg each of HPV-16 and HPV-18 L1 proteins in a 0-, 1-, 6-month schedule  2) HAV vaccine: 360 ELISA units inactivated HAV antigen | 2,067  1) n=1,035  2) n=1,032 | 12 months | Reported SAEs between months 7 and 12, n  1) 13  2) 10  One SAE in the control group was autoimmune thyroiditis.  No AID were reported in the HPV-16/18 vaccine group |
| Naud, 2014  NCT00196924 | Brazil | 1) HPV-16/18 vaccine at 0, 1, and 6 mo  2) placebo (Al[OH]_3_) at 0, 1, and 6 mo | 437  1) n=224  2) n=213 | 36 months | Women reporting hypothyroidism between 77 mo and up to 113 mo post initial vaccination, % (95% CI); n  1) 0.9 (0.1-3.2); 2  2) 0.5 (0.0-2.6); 1 |
| AID: autoimmune disease; Al[OH]_3_: Aluminium hydroxide; CI: Confidence Interval; ELISA: enzyme-linked immunosorbent assay; HAV: hepatitis A virus; HPV: Human papillomavirus; mo: months; NOAD: new-onset autoimmune disease; SAE: serious adverse event | | | | | |

All studies were sponsored or funded by GSK

## Grey and other literature search

In total, 243 trials on cervical cancer vaccine were found in the NIH clinical trial database. Eight studies were found on a bivalent HPV vaccine and still ongoing or recruiting subjects.

One study is an extended follow-up (total of up to 10 years of follow-up) of young women in Costa Rica who received vaccination against HPV 16 and 18 and unvaccinated controls (NCT00867464). Women who were originally in the control arm were offered the HPV-16/18 vaccine at crossover and will also be invited for additional follow-up.

In four studies, subjects were followed after vaccination with Cervarix (NCT00779766, NCT01190176, NCT01190189, NCT01249365). These studies are sponsored by GSK. One study is active, but not recruiting. Three studies are still recruiting subjects.

The other three studies were from a Chinese pharmaceutical company (NCT01735006, NCT01356823, NCT02562508).

The report on autoimmune disease after HPV vaccine by the Agence Nationale de sécurité du Médicament et des produits de santé’ did not yield any new references.

# Discussion

## Discussion of the results

Studies were searched for any report on the occurrence of autoimmune thyroiditis, inflammatory bowel diseases, or Guillain Barré Syndrome in the follow-up period after Cervarix vaccination.

Three studies reported cases of one of these NOAD of interest after vaccination with Cervarix. The follow-up period ranged from twelve months to 36 months. In the first study, all participants received the HPV vaccine in various quantities and/or schedules. Ten subjects reported NOAD, consisting of thyroid disorders and one case each of diabetes mellitus, celiac disease and reactive arthritis. In the second study, no AIDs were reported in the HPV-16/18 vaccine group while in the control group one case of autoimmune thyroiditis was reported. In the third study, two cases of hypothyroidism were reported in the HPV-vaccine group and one case of hypothyroidism in the control group. All studies were funded or sponsored by GSK.

For the additional search on studies evaluating Cervarix but without mentioning AID in the abstract, no evidence tables were made as all relevant studies were funded or sponsored by GSK

Eight studies were found in the NIH clinical trial database on bivalent HPV vaccine of which four studies were sponsored by GSK. All studies were still ongoing or recruiting.

## Limitations of the studies

- Many studies reported number of SAE, NOCD or NOAD in general and did not specify AID.
- In some studies, the authors described only vaccine-related AID instead of giving an overview of all AID.
- Many studies were too small to detect rare AID, i.e. autoimmune thyroiditis, inflammatory bowel diseases or Guillain Barré Syndrome.

Abbreviations

| AID | Autoimmune disease |
| --- | --- |
| Al[OH]3 | Aluminium hydroxide |
| ASIA | Auto-inflammatory Syndrome induced by Adjuvants |
| CD | Crohn’s disease |
| CHMP | Committee for Medicinal Products for Human Use |
| CI | Confidence Interval |
| ELISA | Enzyme-linked immunosorbent assay |
| GBS | Guillain Barré Syndrome |
| HAV | Hepatitis A virus |
| HPV | Human papillomavirus |
| IBD | Inflammatory bowel diseases, |
| MAH | marketing authorisation holder |
| mg | Milligram |
| mo | Months |
| NIH | National Institutes of Health |
| NOAD | New-onset autoimmune disease |
| NOCD | New-onset chronic disease |
| SAE | Serious adverse event |
| SIGN | Scottish Intercollegiate Guidelines Network |
| UC | Ulcerative colitis |
| US | United States |
| VLP | Virus-like particles |

References

1. Miltz A, Price H, Shahmanesh M, et al. Systematic review and meta-analysis of L1-VLP-based human papillomavirus vaccine efficacy against anogenital pre-cancer in women with evidence of prior HPV exposure. PLoS One 2014;**9**(3):e90348.

2. Schiller JT, Castellsague X, Garland SM. A review of clinical trials of human papillomavirus prophylactic vaccines. Vaccine 2012;**30 Suppl 5**:F123-38.

3. Angelo MG, David MP, Zima J, et al. Pooled analysis of large and long-term safety data from the human papillomavirus-16/18-AS04-adjuvanted vaccine clinical trial programme. Pharmacoepidemiology and drug safety 2014;**23**(5):466-79.

4. Rivera Medina DM, Valencia A, de Velasquez A, et al. Safety and Immunogenicity of the HPV-16/18 AS04-Adjuvanted Vaccine: A Randomized, Controlled Trial in Adolescent Girls. Journal of Adolescent Health 2010;**46**(5):414-21.

5. Romanowski B, Schwarz TF, Ferguson LM, et al. Immunogenicity and safety of the HPV-16/18 AS04-adjuvanted vaccine administered as a 2-dose schedule compared with the licensed 3-dose schedule: Results from a randomized study. Human vaccines 2011;**7**(12):1374-86.

6. Naud PS, Roteli-Martins CM, De Carvalho NS, et al. Sustained efficacy, immunogenicity, and safety of the HPV-16/18 AS04-adjuvanted vaccine: final analysis of a long-term follow-up study up to 9.4 years post-vaccination. Human vaccines & immunotherapeutics 2014;**10**(8):2147-62.

1. SIGN Checklists^[[1]](#footnote-1)^

| **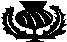 S I G N** | | **Methodology Checklist 2: Controlled Trials** | | | |
| --- | --- | --- | --- | --- | --- |
| Study identification (*Include author, title, year of publication, journal title, pages*) | | | | | |
| Guideline topic: | | | Key Question No: | | Reviewer: |
| **Before** completing this checklist, consider:   1. Is the paper a **randomised controlled trial** or a **controlled clinical trial**? If in doubt, check the study design algorithm available from SIGN and make sure you have the correct checklist. If it is a **controlled clinical trial** questions 1.2, 1.3, and 1.4 are not relevant, and the study cannot be rated higher than 1+ 2. Is the paper relevant to key question? Analyse using PICO (Patient or Population Intervention Comparison Outcome). IF NO REJECT (give reason below). IF YES complete the checklist. | | | | | |
| Reason for rejection: 1. Paper not relevant to key question □ 2. Other reason □ (please specify): | | | | | |
| **Section 1: Internal validity** | | | | | |
| ***In a well conducted RCT study…*** | | | 1. ***Does this study do it?*** | | |
| 1.1 | The study addresses an appropriate and clearly focused question. | | Yes □  Can’t say □ | | No □ |
| 1.2 | The assignment of subjects to treatment groups is randomised. | | Yes □  Can’t say □ | | No □ |
| 1.3 | An adequate concealment method is used. | | Yes □  Can’t say □ | | No □ |
| 1.4 | The design keeps subjects and investigators ‘blind’ about treatment allocation. | | Yes □  Can’t say □ | | No □ |
| 1.5 | The treatment and control groups are similar at the start of the trial. | | Yes □  Can’t say □ | | No □ |
| 1.6 | The only difference between groups is the treatment under investigation. | | Yes □  Can’t say □ | | No □ |
| 1.7 | All relevant outcomes are measured in a standard, valid and reliable way. | | Yes □  Can’t say □ | | No □ |
| 1.8 | What percentage of the individuals or clusters recruited into each treatment arm of the study dropped out before the study was completed? | |  | | |
| 1.9 | All the subjects are analysed in the groups to which they were randomly allocated (often referred to as intention to treat analysis). | | Yes □  Can’t say □ | | No □  Does not apply □ |
| 1.10 | Where the study is carried out at more than one site, results are comparable for all sites. | | Yes □  Can’t say □ | | No □  Does not apply □ |
| **Section 2: OVERALL ASSESSMENT OF THE STUDY** | | | | | |
| 2.1 | How well was the study done to minimise bias?  Code as follows: | | | High quality (++) □  Acceptable (+) □  Low quality (-) □  Unacceptable – reject (0) □ | |
| 2.2 | Taking into account clinical considerations, your evaluation of the methodology used, and the statistical power of the study, are you certain that the overall effect is due to the study intervention? | | |  | |
| 2.3 | Are the results of this study directly applicable to the patient group targeted by this guideline? | | |  | |
| 2.4 | **Notes.** Summarise the authors’ conclusions. Add any comments on your own assessment of the study, and the extent to which it answers your question and mention any areas of uncertainty raised above. | | | | |

| **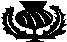 S I G N** | | **Methodology Checklist 3: Cohort studies** | | | | |
| --- | --- | --- | --- | --- | --- | --- |
| Study identification (*Include author, title, year of publication, journal title, pages*) | | | | | | |
| Guideline topic: | | | Key Question No: | | Reviewer: | |
| **Before** completing this checklist, consider:   1. Is the paper really a cohort study? If in doubt, check the study design algorithm available from SIGN and make sure you have the correct checklist. 2. Is the paper relevant to key question? Analyse using PICO (Patient or Population Intervention Comparison Outcome). IF NO REJECT (give reason below). IF YES complete the checklist.. | | | | | | |
| Reason for rejection: 1. Paper not relevant to key question □ 2. Other reason □ (please specify):  **Please note that a retrospective study (ie a database or chart study) cannot be rated higher than +.** | | | | | | |
| **Section 1: Internal validity** | | | | | | |
| ***In a well conducted cohort study:*** | | | | 1. ***Does this study do it?*** | | |
| 1.1 | The study addresses an appropriate and clearly focused question. | | | Yes □  Can’t say □ | | No □ |
| Selection of subjects | | | | | | |
| 1.2 | The two groups being studied are selected from source populations that are comparable in all respects other than the factor under investigation. | | | Yes □  Can’t say □ | | No □  Does not apply □ |
| 1.3 | The study indicates how many of the people asked to take part did so, in each of the groups being studied. | | | Yes □ | | No □  Does not apply □ |
| 1.4 | The likelihood that some eligible subjects might have the outcome at the time of enrolment is assessed and taken into account in the analysis. | | | Yes □  Can’t say □ | | No □  Does not apply □ |
| 1.5 | What percentage of individuals or clusters recruited into each arm of the study dropped out before the study was completed. | | |  | | |
| 1.6 | Comparison is made between full participants and those lost to follow up, by exposure status. | | | Yes □  Can’t say □ | | No □  Does not apply □ |

| ASSESSMENT | | | |
| --- | --- | --- | --- |
| 1.7 | The outcomes are clearly defined. | Yes □  Can’t say □ | No □ |
| 1.8 | The assessment of outcome is made blind to exposure status. If the study is retrospective this may not be applicable. | Yes □  Can’t say □ | No □  Does not apply □ |
| 1.9 | Where blinding was not possible, there is some recognition that knowledge of exposure status could have influenced the assessment of outcome. | Yes □  Can’t say □ | No □ |
| 1.10 | The method of assessment of exposure is reliable. | Yes □  Can’t say □ | No □ |
| 1.11 | Evidence from other sources is used to demonstrate that the method of outcome assessment is valid and reliable. | Yes □  Can’t say □ | No □  Does not apply □ |
| 1.12 | Exposure level or prognostic factor is assessed more than once. | Yes □  Can’t say | No □  Does not apply |
| CONFOUNDING | | | |
| 1.13 | The main potential confounders are identified and taken into account in the design and analysis. | Yes □  Can’t say □ | No □ |
| STATISTICAL ANALYSIS | | | |
| 1.14 | Have confidence intervals been provided? | Yes □ | No □ |
| **Section 2: OVERALL ASSESSMENT OF THE STUDY** | | | |
| 2.1 | How well was the study done to minimise the risk of bias or confounding? | High quality (++) □  Acceptable (+) □  Low quality (-) □  Unacceptable – reject (0) □ | |
| 2.2 | Taking into account clinical considerations, your evaluation of the methodology used, and the statistical power of the study, do you think there is clear evidence of an association between exposure and outcome? | Yes □  Can’t say □ | No □ |
| 2.3 | Are the results of this study directly applicable to the patient group targeted in this guideline? | Yes □ | No □ |
| 2.4 | **Notes.** Summarise the authors conclusions. Add any comments on your own assessment of the study, and the extent to which it answers your question and mention any areas of uncertainty raised above. | | |
|  |  | | |

| **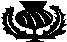 S I G N** | | **Methodology Checklist 4: Case-control studies** | | |
| --- | --- | --- | --- | --- |
| Study identification (*Include author, title, year of publication, journal title, pages*) | | | | |
| Guideline topic: | | | Key Question No: | Reviewer: |
| **Before** completing this checklist, consider:   1. Is the paper really a case-control study? If in doubt, check the study design algorithm available from SIGN and make sure you have the correct checklist. 2. Is the paper relevant to key question? Analyse using PICO (Patient or Population Intervention Comparison Outcome). IF NO REJECT (give reason below). IF YES complete the checklist. | | | | |
| Reason for rejection: Reason for rejection: 1. Paper not relevant to key question □ 2. Other reason □ (please specify): | | | | |
| **Section 1: Internal validity** | | | | |
| ***In an well conducted case control study:*** | | | 1. ***Does this study do it?*** | |
| 1.1 | The study addresses an appropriate and clearly focused question. | | Yes □  Can’t say □ | No □ |
| Selection of subjects | | | | |
| 1.2 | The cases and controls are taken from comparable populations. | | Yes □  Can’t say □ | No □ |
| 1.3 | The same exclusion criteria are used for both cases and controls. | | Yes □  Can’t say □ | No □ |
| 1.4 | What percentage of each group (cases and controls) participated in the study? | | Cases:  Controls: | |
| 1.5 | Comparison is made between participants and non-participants to establish their similarities or differences. | | Yes □  Can’t say □ | No □ |
| 1.6 | Cases are clearly defined and differentiated from controls. | | Yes □  Can’t say □ | No □ |
| 1.7 | It is clearly established that controls are non-cases. | | Yes □  Can’t say □ | No □ |
| ASSESSMENT | | | | |
| 1.8 | Measures will have been taken to prevent knowledge of primary exposure influencing case ascertainment. | | Yes □  Can’t say □ | No □  Does not apply □ |
| 1.9 | Exposure status is measured in a standard, valid and reliable way. | | Yes □  Can’t say □ | No □ |
| CONFOUNDING | | | | |
| 1.10 | The main potential confounders are identified and taken into account in the design and analysis. | | Yes □  Can’t say □ | No □ |
| STATISTICAL ANALYSIS | | | | |
| 1.11 | Confidence intervals are provided. | | Yes □ | No □ |
| **Section 2: OVERALL ASSESSMENT OF THE STUDY** | | | | |
| 2.1 | How well was the study done to minimise the risk of bias or confounding? | | High quality (++) □  Acceptable (+) □  Low quality (-) □  Unacceptable – reject (0) □ | |
| 2.2 | Taking into account clinical considerations, your evaluation of the methodology used, and the statistical power of the study, do you think there is clear evidence of an association between exposure and outcome? | | Yes □  Can’t say □ | No □ |
| 2.3 | Are the results of this study directly applicable to the patient group targeted by this guideline? | | Yes □ | No □ |
| 2.4 | **Notes.** Summarise the authors conclusions. Add any comments on your own assessment of the study, and the extent to which it answers your question and mention any areas of uncertainty raised above.. | | | |

1. Evidence tables

| **Reference**  **Country**  **NCT registration number** | **Setting**  **Study design**  **Study period** | **Population**  **Sample size**  **Follow-up period** |
| --- | --- | --- |
| Romanowski B., Human Vaccines, 2011  Canada, Germany  NCT00541970 | **Setting:** Twenty-one centers in Canada and Germany  **Study design**: Phase I/II, partially-blind, controlled, randomized, age-stratified, parallel group trial  **Study period**: October 2007 – May 2010 | **Population**: Healthy girls and young women aged 9 to 25 years at the time of first vaccination  **Exclusion criteria:** Autoimmune disease or immunodeficiency, more than 14d of immune-suppressants of immune-modifying drugs within 6 mo prior to the first vaccine dose, previously received HPV vaccine, AS04 adjuvant or 3-*O*-desacy-4’-monophosphoryl lipid A, pregnant or breast-feeding  **Sample size**: Enrolled n=961, randomized and vaccinated n=960 of which n=845 had a follow-up of 24 months  Participants were randomized to one of the four groups:  -3-dose 20/20 M0,1,6: n=239  -2-dose 20/20 M0,6: n=240  -2-dose 40/40 M0,6: n=241  -2-dose 40/40 M0,2: n=240 |

| **Vaccine dose**  **Follow-up**  **Outcome detection and definition** | **Results** | **Comments** |
| --- | --- | --- |
| **Vaccine dose:**   - Three doses of HPV-16/18 (20 μg/20 μg) vaccine at Months 0, 1 and 6 (i.e., the licensed vaccine formulation and schedule) (Group 20/20F M0,1,6) - Two doses of HPV-16/18 (20 μg/20 μg) vaccine at Months 0 and 6 (Group 20/20F M0,6) - Two doses of HPV-16/18 (40 μg/40 μg) vaccine at Months 0 and 6 (Group 40/40F M0,6) - Two doses of HPV-16/18 (40 μg/40 μg) vaccine at Months 0 and 2 (Group 40/40F M0,2)   **Follow-up:** 24 months  **Outcome detection and definition**: NOAD occurring through month 24 were documented. NOAD (potential autoimmune events, which excluded allergy-related events or isolated signs and symptoms) were identified by comparing all reported AEs with a pre-defined list of potential chronic diseases derived from the Medical Dictionary for Regulatory Activities | Subjects with new onset of autoimmune-disease, % (n)  3-dose 20/20 M0,1,6: 1.3% (3)  2-dose 20/20 M0,6: 1.3% (3)  2-dose 40/40 M0,6: 0.8% (2)  2-dose 40/40 M0,2: 0.8% (2)  Reported new onset autoimmune diseases consisted of thyroid disorders and one case each of diabetes mellitus, celiac disease and reactive arthritis | - Not clear in which groups thyroid disorders occurred - Relatively small sample size - Trial was partially blinded within the 2-dose schedule group (observers were blinded to group assignment) and open in 3-dose schedule group - No main institute as 21 centres were involved - Limited information on documentation NOAD |
| AE: adverse events; HPV: human papillomavirus; M/mo: month; NOAD: new onset auto-immune disease | | |

| **Reference**  **Country**  **NCT registration number** | **Setting**  **Study design**  **Study period** | **Population**  **Sample size**  **Follow-up period** |
| --- | --- | --- |
| Rivera Medina D., Journal of adolescent health, 2010  Australia, Colombia, the Czech Republic, France, Germany, Honduras, Korea, Norway, Panama, Spain, Sweden, Taiwan  NCT00196924 | **Setting:** Fifty-seven centers in Australia, Colombia, the Czech Republic, France, Germany, Honduras, Korea, Norway, Panama, Spain, Sweden, Taiwan  **Study design**: Phase III, observer-blind, multicenter, randomized, parallel group, controlled study  **Study period**: June 2004 – August 2005 | **Population**: Healthy girls aged 10–14 years  **Exclusion criteria:** Immunodeficiency, history of allergic disease likely to be exacerbated by a vaccine  component, known acute or chronic clinically significant neurologic, hepatic, or renal functional abnormality, history of chronic conditions requiring treatment, or acute disease at enrolment  **Sample size**: Enrolled n=2,067, compliance with 3-dose schedule was 98.2% of which n=2,023 had a follow-up of 12 months  Participants were randomized to one of the two groups:  -HPV-16/18 vaccine: n=1,035  -Control vaccine (HAV vaccine): n=1,032 |

| **Vaccine dose**  **Follow-up**  **Outcome detection and definition** | **Results** | **Comments** |
| --- | --- | --- |
| **Vaccine dose:**   - HPV-16/18 vaccine: 20mg each of HPV-16 and HPV-18 L1 proteins, self-assembled as virus like particles (VLP), adjuvanted with the Adjuvant System AS04 (comprising 500 mg of aluminum hydroxide and 50 mg of the immunostimulatory molecule, 3-O-desacyl-4’ monophosphoryl lipid A) in a 0-, 1-, 6-month schedule - HAV vaccine: 360 ELISA units inactivated HAV antigen and 250 mg aluminum as aluminum hydroxide   **Follow-up:** At months 0, 1, 2, 6, 7 with a telephone call at month 12  **Outcome detection and definition**: SAE, reported up to month 12 | Between months 7 and 12, 13 girls and 10 girls reported SAEs in the HPV-16/18 vaccine and control groups, respectively. One SAE in the control group was autoimmune thyroiditis.  No AID were reported in the HPV-16/18 vaccine group | - Limited information on documentation SAE - Not insight in the SAE reported up to month 7 - No main institute as 57 centres were involved - Small sample size - Concealment method unclear |
| AID: autoimmune disease; ELISA: enzyme-linked immunosorbent assay; HAV: hepatitis A virus; HPV: human papillomavirus; mg: milligram; SAE: serious adverse event. | | |

| **Reference**  **Country**  **NCT registration number** | **Setting**  **Study design**  **Study period** | **Population**  **Sample size**  **Follow-up period** |
| --- | --- | --- |
| Naud P., Human vaccines & immunotherapeutics, 2014  Brazil  NCT00196924 | **Setting:** Five hospital-based Brazilian centers  **Study design**: Follow-up of an initial double-blind, randomized, multi-center vaccination study  **Study period**: November 2007-2010 | **Population**: Subset of women enrolled in HPV-001 and who participated in the follow-up study HPV-007 whose treatment allocation had remained blinded in both studies. For HPV-001, healthy women aged 15-25 y were recruited. Women were HPV-16 and HPV-18 seronegative by ELISA, HPV DNA-negative in the cervix by PCR for 14 oncogenic types (HPV-16,-18,-31,-33,-35,- 39,-45,-51,-52,-56,-58,-59,-66,-68),and had normal cervical cytology at baseline. Mean age at HPV-023 study entry was 26.5y (SD 3.1y)  **Exclusion criteria:** NR  **Sample size**: Enrolled in HPV-001 n=506, 448 continued into HPV-007, n=437 agreed to continue in HPV-023 of which n=431 completed the study  Participants were randomized to one of the two groups:  -HPV-16/18 vaccine: n=224  -Placebo (Al[OH]_3_): n=213 |

| **Vaccine dose**  **Follow-up**  **Outcome detection and definition** | **Results** | **Comments** |
| --- | --- | --- |
| **Vaccine dose:**   - 3 doses of HPV-16/18 vaccine at 0, 1, and 6 mo - 3 doses of placebo (Al[OH]_3_) at 0, 1, and 6 mo   **Follow-up:** Three years, with data collected from end of HPV-007 up to the final visit (month 36) in HPV-023. Mean follow-up time since first vaccination in HPV-001 was 107 months (8.9y, SD=0.4)  **Outcome detection and definition**: NOCDs (e.g., NOADs, asthma, type I diabetes) were recorded | Number and percentage of women reporting NOAD between 77 mo and up to 113 mo post initial vaccination (36-mo follow-up), % (95% CI); n   \|  \| Vaccine (N=224) \| Placebo (N=213) \| \| --- \| --- \| --- \| \|  \| % (95% CI); n \| % (95% CI); n \| \| NOAD in general \| 1.8 (0.5-4.5); 4 \| 0.5 (0.0-2.6); 1 \| \| - Hypothyroidism \| 0.9 (0.1-3.2); 2 \| 0.5 (0.0-2.6); 1 \| | - Concealment method unclear - Unclear if subjects and investigators were kept blind about treatment allocation - Small sample size - No main institute mentioned |
| CI: confidence interval; DNA: deoxyribonucleic acid; ELISA: enzyme-linked immunosorbent assay; HPV: human papillomavirus; mo: month; NOAD: new onset autoimmune disease; NOCD: new onset chronic disease; NR: not reported; PCR polymerase chain reaction; SD: standard deviation; y: year. | | |

1. List of studies reporting number of NOAD, but with unclear number of the AID of interest (original search)

|  | **ClinicalTrials.gov Identifier** | **Reference** | **Reported outcomes** | **GSK study^1^** | **Country** | **Follow-up period** |
| --- | --- | --- | --- | --- | --- | --- |
| 1 | Safety data from 11 Phase II/III trials | Descamps_ Hum Vaccin_ 2009 | The overall percentage of women reporting NOADs was 0.3% (95% CI: 0.1, 0.9) in the HPV-16/18 vaccine group 10-14 yrs, 0.4% (95% CI:0.3-0.5) in the HPV-16/18 vaccine group15-24 yrs - "The most frequent NOADs were related to thyroid disease" | yes | 30 countries  in North and Latin America, Europe, Australia and Asia | -* |
| 2 | NCT00423046 | Einstein_Hum Vaccin Immunother_ 2014 | 102 SAE, 66 subjects experienced NOCDs, 19 of these subjects reported NOCDs that were identified as NOADs. The most common NOCD/NOAD was hypothyroidism. | yes | US | 48 mo |
| 3 | NCT00423046 | Einstein_ Hum Vaccin Immunother_ 2014_2 | 20 NOAD, The most commonly identified NOCD and NOAD was hypothyroidism | yes | US | 60 mo |
| 4 | NCT01462357 | Leung_ Human Vaccines and Immunotherapeutic_ 2015 | 6 potential immune-mediated diseases. The reported pIMDs were reactive arthritis, juvenile idiopathic arthritis, erythema nodosum, alopecia areata, ulcerative colitis and celiac disease** | yes | France, Hong Kong, Singapore and Sweden | 12 mo |
| 5 | NCT00689741 (NCT00518336/ NCT00120848) | Roteli-Martins_Hum Vaccin Immunother_2012 | 17 SAE, 7 NOCD, 2 NOAD, of which 1 case of hypothyroidism*** | yes | (US, Canada) Brazil | 8,4 y |
| 6a | NCT00122681 | Paavonen_Lancet_2007 | 57 NOAD, no mentioning of AID of interest | yes | 14 countries in Asia Pacific, Europe, Latin America, and North America. | 14,8 mo |
| 6b | NCT00122681 | Lehtinen_Lancet Oncol_2012 | 194 NOAD, no mentioning of AID of interest | Yes | 14 countries in Asia Pacific, Europe, Latin America, and North America | 48 mo |

AID of interest: autoimmune thyroiditis, inflammatory bowel disease, or Guillain Barré Syndrome

1: study sponsored or funded by GSK; *pooled results from 11 trials, including safety results from 0-30 days post-vaccination, month 0-7, month 7-12 and post month 12. **Not clear in which treatment group *** Not clear of NOAD cases in vaccinated or placebo-group occured. "cases remain blinded with respect to treatment allocation as the study is still ongoing"

AID: autoimmune diease; mo: months; NOAD: new-onset autoimmune diease; NOCD: new-onset chronic disease; SAE: serious adverse event; y: years

1. List additional search

**Number of serious adverse events reported, including the number of autoimmune disease cases**

|  | **ClinicalTrials.gov Identifier** | **Reference** | **Reported outcomes** | **GSK study^1^** | **Country** | **Follow-up period** |
| --- | --- | --- | --- | --- | --- | --- |
| 1 | NCT 00485732 | Kim_J Gynecol Oncol_2011 | 3 SAE, 0 cases of AID* | Yes | Korean | 7 mo |
| 2 | NCT00169494 | Pedersen_J Adolesc Health_2007 | 8 SAE, 0 cases of AID* | Yes | Denmark, Estonia, Finland, Greece, The Netherlands, and Russia | 7 mo |
| 3 | NCT00290277 | Kim_J Korean Med Sci_2010 | 1 SAE, 0 cases of AID* | Yes | Korean | 7 mo |
| 4 | NCT00306241 | Ngan_Hong Kong Med J_2010 | 4 SAE, 0 cases of AID* | Yes | China/Hong Kong | 7 mo |
| 5 | NCT00316693 | Konno_Int J Gynecol Cancer_2009 | 16 SAE, 0 cases of AID* | Yes | Japan | 7 mo |
| 6 | NCT00344032 | Bhatla_J Obstet Gynaecol Res_2010 | 6 SAE, 0 cases of AID* | Yes | India | 7 mo |
| 7 | NCT00345878 | Lim_Med J Malaysia_2014 | 8 SAE, 0 cases of AID* | Yes | Malaysia | 7 mo |
| 8 | NCT00426361 | Garcia-Sicilia_J Adolesc Health_2010 | 12 SAE, 0 cases of AID* | Yes | France, Germany, and Spain | 7-8 mo |
| 9 | NCT00578227 | Pedersen-J Adolesc Health-2012 | 11 SAE, 0 cases of AID* | Yes | Canada, Denmark, Hungary, and Sweden | 7 mo |
| 10 | NCT00689741; (initial study) (NCT00120848) NCT00518336; (current follow-up study) | De Carvalho_Vaccine_2010 | 9 SAE, 0 cases of AID* | Yes | Brazil | 7.3 y |
| 11 | NCT00996125/ NCT01277042 | Zhu-Hum Vaccin Immunother-2014 | 2 NOAD, 0 cases in HPV group | Yes | China | 7 mo |
| 12 | NCT00128661 | Hildesheim_Vaccine_2014 | 43 NOAD, of which: goiter (8 in HPV arm; 9 in control arm); ), inflammatory bowel dis-ease (3 in HPV arm including 1 Crohn’s disease; 2 in control arm) [other conditions (4 in HPV arm; 2 in control arm).] 15 death, Crohn’s disease (1 in HPV arm) | Yes |  | 4 y |
| 13 | No trial number | Brabin_Bmj_2008 | No SAE | Yes | UK | Unclear |

1: study sponsored or funded by GSK; 2: vaccine at 0, 1, and 6 months (although they mention only two doses) and a process for reporting serious adverse events; *AID of interest: autoimmune thyroiditis, inflammatory bowel disease, or Guillain Barré Syndrome ; AID: autoimmune disease; HPV: human papillomavirus; mo: months; NOAD: new-onset autoimmune disease; SAE: serious adverse event; y: years

**Number of serious adverse events mentioned, but not clear if autoimmune disease were present**

|  | **ClinicalTrials.gov Identifier** | **Reference** | **Reported outcomes** | **GSK study^1^** | **Country** | **Follow-up period** |
| --- | --- | --- | --- | --- | --- | --- |
| 1a | NCT00196924 (NCT00316706) | Schwarz_J Adolesc Health_2012 | 76 SAE, no description of AID* | Yes | Taiwan, Germany, Honduras, Panama, and Colombia | 48 mo |
| 1b | NCT00196924 (NCT00877877/ NCT00316706) | Schwarz_Pediatr Infect Dis J_2014 | 110 SAE, no description of AID* | Yes | Taiwan, Germany, Honduras, Panama and Colombia | 72 mo |
| 2a | NCT00196937 (105879/014) | Schwarz_Vaccine_2009 | 15 SAE, no description of AID* | Yes | Germany and Poland | 24 mo |
| 2b | NCT00196937 (105882) | Schwarz-Hum Vaccin-2011 | 29 SAE, of which 14 NOCD, no description of AID* | Yes | Germany and Poland | 48 mo |
| 2c | NCT00196937 (NCT00947115 ) | Schwarz-Bjog-2015 | 32 SAE, no description of AID* | Yes | Germany and Poland | 72 mo |
| 3a | NCT00316693 | Konno_Int J Gynecol Cancer_2010 (interim analysis) | 37 SAE, no description of AID* | Yes | Japan | 13.6 mo |
| 3b | NCT00316693 | Konno_Int J Gynecol Cancer_2010 | 37 SAE, 11 NOCD, no description of AID* | Yes | Japan | 24 mo |
| 4 | NCT00689741 Current study: NCT00546078 | Moscicki_Vaccine_2012 | No mentioning of SAE or NOCD (description in methods of safety assessment) | Yes | US, Canada, Brazil | 7 days or 7 mo |
| 5 | No trial number | Khatun_Jpn J Clin Oncol_2012 | Unclear description, no NOAD | No | Bangladesh | 7 mo |

*AID of interest: autoimmune thyroiditis, inflammatory bowel disease, or Guillain Barré Syndrome; 1: study sponsored or funded by GSK;

AID: autoimmune disease; mo: months; NOAD: new-onset autoimmune disease; SAE: serious adverse event; y: years

**NOAD in general, disease not further specified**

|  | **ClinicalTrials.gov Identifier** | **Reference** | **Reported outcome** | **GSK study^1^** | **Country** | **Follow-up period** |
| --- | --- | --- | --- | --- | --- | --- |
| 1 | NCT00294047 | Skinner_Lancet_2014 | 13 NOAD, not further specified | Yes | Australia, Canada, Mexico, the Netherlands, Peru, Philippines, Portugal, Russia, Singapore, Thailand, UK, and US | 48 mo |
| 2 | NCT00337818 | Petaja-Int J Cancer-2011 | 168 medically significant AEs, 23 NOCD, the most frequently reported NOCDs were asthma (5) and hypothyroidism (3) | yes | Denmark, Estonia and Finland | 48 mo |
| 3a | NCT00423046 | Einstein_Hum Vaccin_2009 | NOCD were observed in 14 women in the Cervarix™ group and 13 in the Gardasil ® group. The most frequent were depression, hypertension, and hypothyroidism. Four NOCD cases were considered to be NOAD. | Yes | US | 7 mo |
| 3b | NCT00423046 | Einstein_Hum Vaccin_2011 | NOAD in Cervarix group 1.1% (0.4-2.3) and HPV 6/11/16/18 group 1.8% (0.9-3.3), not further specified | Yes | US | 24 mo |
| 4 | NCT00481767 | Sow-J Infect Dis-2013 | 4 NOAD, 2 in vaccine group, not further specified | Yes | Senegal, Tanzania | 12 mo |
| 5a | NCT00541970 | Romanowski_Hum Vaccin Immunother_2014 | 10 NOAD, not further specified | Yes | Canada, Germany | 48 mo |
| 5b | NCT00541970 | Romanowski_Hum Vaccin Immunother_2015 | 11 NOAD, not further specified | Yes | Canada, Germany | 60 mo |
| 6 | NCT00779766 | Zhu-Int J Cancer-2014 | 4 NOAD, 2 in vaccine group, not further specified | Yes | China | 15,3 mo |
| 7 | NCT00929526 (initial study: NCT00316693) | Konno_Hum Vaccin Immunother_2014 | 4 NOAD, 3 in vaccine group, not further specified | Yes | Japan | 4 y |
| 8 | NCT00122681 | Paavonen_Lancet_2009 | 155 NOAD, not further specified | Yes | 14 countries in Asia Pacific, Europe, Latin America, and North America. | 34,9 mo |

1: study sponsored or funded by GSK

AID: autoimmune disease; HPV: human papillomavirus; mo: months; NOAD: new-onset autoimmune disease; SAE: serious adverse event; y: years

1. SIGN: http://www.sign.ac.uk/methodology/checklists.html [↑](#footnote-ref-1)
