# Supplementary material for: Meta‐analysis of the risk of autoimmune thyroiditis, Guillain‐Barré syndrome, and inflammatory bowel disease following vaccination with AS04‐adjuvanted human papillomavirus 16/18 vaccine
Source: Pharmacoepidemiol Drug Saf. 2020 Jun 24;29(9):1159–67. doi: 10.1002/pds.5063 (PMC7539912; doi:10.1002/pds.5063)
Supplement: Supplementary file 1 — Data S1. Supporting Information. [file PDS-29-1159-s001.zip › PDS_5063_pds-19-0290-File003.docx]

**Supporting information B.** RCTs included in the meta-analysis

| Study^ref^ | www.clinicaltrials.gov | Exposed, n | Control, n | Control | Age, years | Country |
| --- | --- | --- | --- | --- | --- | --- |
| HPV-001^1^ +  follow-up: HPV-007†^2^ | NCT00689741/  NCT00518336 | 560 | 553 | Placebo‡ | 15–25 | Canada, USA, Brazil |
| HPV-008^3^ | NCT00122681 | 9328§ | 9337§ | HAV | 15–25 | 14 |
| HPV-009^4^ | NCT00128661 | 3729¶ | 3737¶ | HAV | 18–25 | Costa Rica |
| HPV-013^5^ +  Extension M18 +  Extention M24 | NCT00196924/  NCT00316706/  NCT00877877 | 1035 | 1032 | HAV | 10–14 | 12 |
| HPV-015^6^ | NCT00294047 | 2881 | 2871 | Placebo‡ | 26+ | 12 |
| HPV-020^7^ | NCT00586339 | 91 | 59 | Placebo‡ | 18–25 | South Africa |
| HPV-021^8^ | NCT00481767 | 450 | 226 | Placebo‡ | 10–25 | Africa |
| HPV-026^9^ | NCT00637195 | 76 | 76 | HBV | 20–25 | Belgium |
| HPV-029^10^ | NCT00578227 | 542 | 271 | HAB | 9–15 | Canada, Denmark, Hungary, Sweden |
| HPV-030^11^ | NCT00652938 | 494 | 247 | HBV | 9–15 | The Netherlands, Sweden |
| HPV-031^12^ | NCT00344032 | 176 | 178 | Placebo‡ | 18–35 | India |
| HPV-032^13^ | NCT00316693 | 519 | 521 | HAV | 20–25 | Japan |
| HPV-033^14^ | NCT00290277 | 160 | 161 | HAV | 10–14 | Korea |
| HPV-035^15^ | NCT00306241 | 150 | 150 | Placebo‡ | 18–35 | China |
| HPV-036^16^ | NCT00345878 | 135 | 136 | Placebo‡ | 18–35 | Malaysia |
| HPV-038^17^ | NCT00485732 | 149 | 76 | Placebo‡ | 15–25 | Korea |
| HPV-058^18^ | NCT00996125 | 374 | 376 | Placebo‡ | 9–17 | China |
| HPV-069^18^ | NCT01277042 | 606 | 606 | HBV | 26–45 | China |
| Total | – | 21,455 | 20,613 | – | – | – |

HAB, combined hepatitis A and hepatitis B vaccine; HAV, hepatitis A vaccine; HBV, hepatitis B vaccine; RCT, randomized controlled trial; USA, United States of America; n, number of subjects.

†Limited to 2 years following the first dose.

‡Al(OH)_3_.

§These numbers do not match those in the publications because we included an additional 21 patients who were excluded from the published analysis due to concerns about data integrity.

¶These numbers do not match those in the publications because two patients in the control group in the published analysis received an HPV dose so are included in the exposed cohort in this analysis.

**References**

1. Harper DM, Franco EL, Wheeler C, Ferris DG, Jenkins D, Schuind A, Zahaf T, Innis B, Naud P, De Carvalho NS, Roteli-Martins CM, Teixeira J, Blatter MM, Korn AP, Quint W, Dubin G, GlaxoSmithKline HPV Vaccine Study Group. Efficacy of a bivalent L1 virus-like particle vaccine in prevention of infection with human papillomavirus types 16 and 18 in young women: a randomised controlled trial. *Lancet* 2004; **364**: 1757-1765.

2. Harper DM, Franco EL, Wheeler CM, Moscicki AB, Romanowski B, Roteli-Martins CM, Jenkins D, Schuind A, Costa Clemens SA, Dubin G, HPV Vaccine Study group. Sustained efficacy up to 4.5 years of a bivalent L1 virus-like particle vaccine against human papillomavirus types 16 and 18: follow-up from a randomised control trial. *Lancet* 2006; **367**: 1247-1255.

3. Paavonen J, Naud P, Salmeron J, Wheeler CM, Chow SN, Apter D, Kitchener H, Castellsague X, Teixeira JC, Skinner SR, Hedrick J, Jaisamrarn U, Limson G, Garland S, Szarewski A, Romanowski B, Aoki FY, Schwarz TF, Poppe WA, Bosch FX, Jenkins D, Hardt K, Zahaf T, Descamps D, Struyf F, Lehtinen M, Dubin G, HPV PATRICIA Study Group. Efficacy of human papillomavirus (HPV)-16/18 AS04-adjuvanted vaccine against cervical infection and precancer caused by oncogenic HPV types (PATRICIA): final analysis of a double-blind, randomised study in young women. *Lancet* 2009; **374**: 301-314.

4. Herrero R, Wacholder S, Rodriguez AC, Solomon D, Gonzalez P, Kreimer AR, Porras C, Schussler J, Jimenez S, Sherman ME, Quint W, Schiller JT, Lowy DR, Schiffman M, Hildesheim A, Costa Rica Vaccine Trial Group. Prevention of persistent human papillomavirus infection by an HPV16/18 vaccine: a community-based randomized clinical trial in Guanacaste, Costa Rica. *Cancer Discov* 2011; **1**: 408-419.

5. Medina DM, Valencia A, de Velasquez A, Huang LM, Prymula R, Garcia-Sicilia J, Rombo L, David MP, Descamps D, Hardt K, Dubin G, HPV Study Group. Safety and immunogenicity of the HPV-16/18 AS04-adjuvanted vaccine: a randomized, controlled trial in adolescent girls. *J Adolesc Health* 2010; **46**: 414-421.

6. Skinner SR, Szarewski A, Romanowski B, Garland SM, Lazcano-Ponce E, Salmeron J, Del Rosario-Raymundo MR, Verheijen RH, Quek SC, da Silva DP, Kitchener H, Fong KL, Bouchard C, Money DM, Ilancheran A, Cruickshank ME, Levin MJ, Chatterjee A, Stapleton JT, Martens M, Quint W, David MP, Meric D, Hardt K, Descamps D, Geeraerts B, Struyf F, Dubin G, VIVIANE Study Group. Efficacy, safety, and immunogenicity of the human papillomavirus 16/18 AS04-adjuvanted vaccine in women older than 25 years: 4-year interim follow-up of the phase 3, double-blind, randomised controlled VIVIANE study. *Lancet* 2014; **384**: 2213-2227.

7. Denny L, Hendricks B, Gordon C, Thomas F, Hezareh M, Dobbelaere K, Durand C, Herve C, Descamps D. Safety and immunogenicity of the HPV-16/18 AS04-adjuvanted vaccine in HIV-positive women in South Africa: a partially-blind randomised placebo-controlled study. *Vaccine* 2013; **31**: 5745-5753.

8. Sow PS, Watson-Jones D, Kiviat N, Changalucha J, Mbaye KD, Brown J, Bousso K, Kavishe B, Andreasen A, Toure M, Kapiga S, Mayaud P, Hayes R, Lebacq M, Herazeh M, Thomas F, Descamps D. Safety and immunogenicity of human papillomavirus-16/18 AS04-adjuvanted vaccine: a randomized trial in 10-25-year-old HIV-seronegative African girls and young women. *J Infect Dis* 2013; **207**: 1753-1763.

9. Leroux-Roels G, Haelterman E, Maes C, Levy J, De Boever F, Licini L, David MP, Dobbelaere K, Descamps D. Randomized trial of the immunogenicity and safety of the Hepatitis B vaccine given in an accelerated schedule coadministered with the human papillomavirus type 16/18 AS04-adjuvanted cervical cancer vaccine. *Clin Vaccine Immunol* 2011; **18**: 1510-1518.

10. Pedersen C, Breindahl M, Aggarwal N, Berglund J, Oroszlan G, Silfverdal SA, Szuts P, O'Mahony M, David MP, Dobbelaere K, Dubin G, Descamps D. Randomized trial: immunogenicity and safety of coadministered human papillomavirus-16/18 AS04-adjuvanted vaccine and combined hepatitis A and B vaccine in girls. *J Adolesc Health* 2012; **50**: 38-46.

11. Schmeink CE, Bekkers RL, Josefsson A, Richardus JH, Berndtsson Blom K, David MP, Dobbelaere K, Descamps D. Co-administration of human papillomavirus-16/18 AS04-adjuvanted vaccine with hepatitis B vaccine: randomized study in healthy girls. *Vaccine* 2011; **29**: 9276-9283.

12. Bhatla N, Suri V, Basu P, Shastri S, Datta SK, Bi D, Descamps DJ, Bock HL, Indian HPV Vaccine Study Group. Immunogenicity and safety of human papillomavirus-16/18 AS04-adjuvanted cervical cancer vaccine in healthy Indian women. *J Obstet Gynaecol Res* 2010; **36**: 123-132.

13. Konno R, Tamura S, Dobbelaere K, Yoshikawa H. Efficacy of human papillomavirus type 16/18 AS04-adjuvanted vaccine in Japanese women aged 20 to 25 years: final analysis of a phase 2 double-blind, randomized controlled trial. *Int J Gynecol Cancer* 2010; **20**: 847-855.

14. Kim YJ, Kim KT, Kim JH, Cha SD, Kim JW, Bae DS, Nam JH, Ahn WS, Choi HS, Ng T, Bi D, Ok JJ, Descamps D, Bock HL. Vaccination with a human papillomavirus (HPV)-16/18 AS04-adjuvanted cervical cancer vaccine in Korean girls aged 10-14 years. *J Korean Med Sci* 2010; **25**: 1197-1204.

15. Ngan HY, Cheung AN, Tam KF, Chan KK, Tang HW, Bi D, Descamps D, Bock HL. Human papillomavirus-16/18 AS04-adjuvanted cervical cancer vaccine: immunogenicity and safety in healthy Chinese women from Hong Kong. *Hong Kong Med J* 2010; **16**: 171-179.

16. Lim BK, Ng KY, Omar J, Omar SZ, Gunapalaiah B, Teoh YL, Bock HL, Bi D. Immunogenicity and Safety of the AS04-adjuvanted Human Papillomavirus-16/18 Cervical Cancer Vaccine in Malaysian Women Aged 18-35 years: A Randomized Controlled Trial. *Med J Malaysia* 2014; **69**: 2-8.

17. Kim SC, Song YS, Kim YT, Kim YT, Ryu KS, Gunapalaiah B, Bi D, Bock HL, Park JS. Human papillomavirus 16/18 AS04-adjuvanted cervical cancer vaccine: immunogenicity and safety in 15-25 years old healthy Korean women. *J Gynecol Oncol* 2011; **22**: 67-75.

18. Zhu F, Li J, Hu Y, Zhang X, Yang X, Zhao H, Wang J, Yang J, Xia G, Dai Q, Tang H, Suryakiran P, Datta SK, Descamps D, Bi D, Struyf F. Immunogenicity and safety of the HPV-16/18 AS04-adjuvanted vaccine in healthy Chinese girls and women aged 9 to 45 years. *Hum Vaccin Immunother* 2014; **10**: 1795-1806.
