# Supplementary material for: Meta‐analysis of the risk of autoimmune thyroiditis, Guillain‐Barré syndrome, and inflammatory bowel disease following vaccination with AS04‐adjuvanted human papillomavirus 16/18 vaccine
Source: Pharmacoepidemiol Drug Saf. 2020 Jun 24;29(9):1159–67. doi: 10.1002/pds.5063 (PMC7539912; doi:10.1002/pds.5063)
Supplement: Supplementary file 1 — Data S1. Supporting Information. [file PDS-29-1159-s001.zip › PDS_5063_pds-19-0290-File005.docx]

**Supporting information D.** Clinical definitions used in the studies

| Event | Pooled clinical and cluster-randomized trial^1,2^ | UK cohort study†^3^ | French cohort study^4-6^ | Case–control study^7,8^ |
| --- | --- | --- | --- | --- |
|  | MedDRA Primary System Organ Class and Preferred Term | Read codes + ICD-10 | ICD-10 codes | Study clinical definition |
| AIT | - Basedow's disease (10004161) - AIT (10049046) | Confirmed cases:   - AIT - Basedow’s disease - Graves’ disease - Hashimoto’s disease - De Quervain’s thyroiditis - Riedel's thyroiditis   (E05, E06.1, E06.3, E06.5) | Thyroiditis was defined by the use of specific drugs indicated for the treatment of thyroid  disorders (≥2 dispensations within 6 months) plus ≥1 of:   - Routine thyroid function tests (T3±T4±TSH) combined with complementary examination of the thyroid (imaging tests or autoantibody levels)‡,§ - Hospital stay with an ICD-10 code of thyroiditis¶ as the main diagnosis or a related diagnosis - A new full coverage for thyroiditis as a long-term illness. | AIT: definite case according to study definitions including Graves’ disease and Hashimoto’s disease†† |
| GBS | GBS (10018767) | Confirmed cases of GBS† (G61.0, G60) | One of:   - GBS (G61.0) - Occurrence of ALD30 (long-term diseases) for GBS - ≥1 hospitalization of ≥6 days with a principal diagnosis of stay or related diagnosis for GBS - ≥2 hospitalizations with a principal diagnosis of stay or related diagnosis for GBS | GBS definite case (level 1) according to the Brighton collaboration case definition:^9^ requires clinical, electrophysiological, and CSF data consistent with the onset of GBS |
| IBD | - Colitis ulcerative (10009900) - Crohn’s disease (10011401) - Proctitis ulcerative (10036783) - IBD (10021972) [not for HPV-040] | Confirmed cases of   - Crohn’s diseases (K50) - Ulcerative colitis (K51) | One of:   - ALD30 (long-term disease) for IBD - ≥1 hospitalization with a principal diagnosis of stay or related diagnosis for IBD   Plus:   - 1 hospitalization for lower gastrointestinal endoscopy before or at the time of diagnosis   (K50, K51) | Not assessed |

AIT, autoimmune thyroiditis; CSF, cerebrospinal fluid; IBD, inflammatory bowel disease; ICD-10, **International Statistical Classification of Diseases and Related Health Problems**, 10th Revision; GBS, Guillain–Barré syndrome; MedDRA, Medical Dictionary for Regulatory Activity; PGRx, Pharmacoepidemiological General Research eXtension; TSH: thyroid-stimulating hormone; TPO, thyroperoxidase; UK, United Kingdom.

†In addition to the specific ICD-10 codes listed in the table, other codes were used to capture possible cases. Among these, only cases confirmed by a medical review were included.

‡Codes: 1206 (free triiodothyronine [FT3]); 1207 (free thyroxine [FT4]); 1208 thyroid-stimulating hormone (TSH) for diagnosis of thyroid dysfunction or thyroid function monitoring; 1209 (FT3 + FT4); 1210 (TSH + FT3); 1211 (TSH + FT4); 1212 (TSH + FT3 + FT4); 1483/4 (anti-thyroglobulin antibodies); 1485/6 (thyroid antimicrosomes antibodies); 1487 (thyroid peroxydase antibodies); 1488 (anti-TSH receptor antibodies).

§Medical procedures: KCHB001 (transcutaneous thyroid fine-needle aspiration cytology without ultrasound guidance); KCHJ001 (transcutaneous thyroid fine-needle aspiration cytology under ultrasound guidance); KCHJ002 (transcutaneous thyroid biopsy [several lesions] under ultrasound guidance); KCHJ003 (transcutaneous thyroid biopsy [one lesion] under ultrasound guidance); KCHJ004 (transcutaneous thyroid fine-needle aspiration cytology from several thyroid lesions under ultrasound guidance); KCQL001 (thyroid scintigraphy with iodine uptake measurement); KCQL002 (thyroid iodine uptake measurement); KCQL003 (thyroid scintigraphy); KCQM001 (thyroid ultrasound).

¶ICD-10 codes: E03.4 Atrophy of thyroid (acquired); E03.5 Myxedema coma; E03.8 Other specified hypothyroidism; E03.9 Hypothyroidism, unspecified; E04.0 Nontoxic diffuse goiter; E04.8 Other specified nontoxic goiter; E04.9 Nontoxic goiter, unspecified; E05.0 Thyrotoxicosis with diffuse goitre; E05.5 Thyroid crisis or storm; E05.8 Other thyrotoxicosis; E05.9 Thyrotoxicosis, unspecified; E06.0 Acute thyroiditis; E06.1 Subacute thyroiditis; E06.2 Chronic thyroiditis with transient thyrotoxicosis; E06.3 Autoimmune thyroiditis; E06.5 Other chronic thyroiditis; E06.9 Thyroiditis, unspecified.

††Definite case of AIT = hypothyroidism consistent with incident autoimmune thyroiditis AND antiperoxydase (anti-TPO) AND increased TSH >7 mU/L; Definite case of Graves’ disease = Presence of exophthalmia or palsy or tachycardio or weight loss or weight gain AND anti-TSH-receptor AND decreased TSH.

**References**

1. Lehtinen M, Apter D, Baussano I, Eriksson T, Natunen K, Paavonen J, Vanska S, Bi D, David MP, Datta S, Struyf F, Jenkins D, Pukkala E, Garnett G, Dubin G. Characteristics of a cluster-randomized phase IV human papillomavirus vaccination effectiveness trial. *Vaccine* 2015; **33**: 1284-1290.

2. GlaxoSmithKline Biologicals. An observational cohort study to assess the risk of autoimmune diseases in adolescent and young adult women aged 9 to 25 years exposed to Cervarix® in the United Kingdom. Study number 116239 (EPI-HPV-040 VS UK). <https://gsk.sylogent.com/files/116239-Clinical-Study-Result-Summary.pdf> (accessed 25 October 2016).

3. Willame C, Rosillon D, Zima J, Angelo MG, Stuurman AL, Vroling H, Boggon R, Bunge EM, Pladevall-Vila M, Baril L. Risk of new onset autoimmune disease in 9- to 25-year-old women exposed to human papillomavirus-16/18 AS04-adjuvanted vaccine in the United Kingdom. *Hum Vaccin Immunother* 2016; **12**: 2862-2871.

4. Agence Nationale de Sécurité du Médicament et des produits de santé (ANSM). Vaccination contre les infections à HPV et risque de maladies auto-immunes : une étude Cnamts/ANSM rassurante - Point d'information. <http://ansm.sante.fr/S-informer/Points-d-information-Points-d-information/Vaccination-contre-les-infections-a-HPV-et-risque-de-maladies-auto-immunes-une-etude-Cnamts-ANSM-rassurante-Point-d-information> (accessed 26 August 2016).

5. Collin C, Miranda S, Zureik M, Dray-Spira R. HPV vaccines and the risk of thyroiditis in girls. Complementary analyses of the French cohort based on data from SNIIRAM. 2018.

6. Miranda S, Chaignot C, Collin C, Dray-Spira R, Weill A, Zureik M. Human papillomavirus vaccination and risk of autoimmune diseases: A large cohort study of over 2 million young girls in France. *Vaccine* 2017; **35**: 4761-4768.

7. Grimaldi-Bensouda L, Rossignol M, Kone-Paut I, Krivitzky A, Lebrun-Frenay C, Clet J, Brassat D, Papeix C, Nicolino M, Benhamou PY, Fain O, Costedoat-Chalumeau N, Courcoux MF, Viallard JF, Godeau B, Papo T, Vermersch P, Bourgault-Villada I, Breart G, Abenhaim L, PGRx-AD Study Group. Risk of autoimmune diseases and human papilloma virus (HPV) vaccines: Six years of case-referent surveillance. *J Autoimmun* 2017; **79**: 84-90.

8. Grimaldi-Bensouda L, Aubrun E, Abenhaim L. ANALYSIS OF CERVARIX® & AUTOIMMUNE DISORDERS USING THE PGRx INFORMATION SYSTEM. Data on file. 2015.

9. Sejvar JJ, Kohl KS, Gidudu J, Amato A, Bakshi N, Baxter R, Burwen DR, Cornblath DR, Cleerbout J, Edwards KM, Heininger U, Hughes R, Khuri-Bulos N, Korinthenberg R, Law BJ, Munro U, Maltezou HC, Nell P, Oleske J, Sparks R, Velentgas P, Vermeer P, Wiznitzer M, Brighton Collaboration GBS Working Group. Guillain-Barré syndrome and Fisher syndrome: case definitions and guidelines for collection, analysis, and presentation of immunization safety data. *Vaccine* 2011; **29**: 599-612.
