# Supplementary material for: Meta‐analysis of the risk of autoimmune thyroiditis, Guillain‐Barré syndrome, and inflammatory bowel disease following vaccination with AS04‐adjuvanted human papillomavirus 16/18 vaccine
Source: Pharmacoepidemiol Drug Saf. 2020 Jun 24;29(9):1159–67. doi: 10.1002/pds.5063 (PMC7539912; doi:10.1002/pds.5063)
Supplement: Supplementary file 1 — Data S1. Supporting Information. [file PDS-29-1159-s001.zip › PDS_5063_pds-19-0290-File006.docx]

**Supporting information E.**

For the French cohort study,^1-3^ only the total numbers of events and the mean follow-up periods were known. Further, events were reported in exposed and non-exposed subjects, which included a combination of non-vaccinated subjects plus the pre-exposure periods of subjects who were subsequently vaccinated with AS04-HPV-16/18 or HPV-6/11/16/18. The number of events occurring among only non-vaccinated subjects was estimated using the total number of events among non-vaccinated and pre-exposed subjects multiplied by the number of person-years of follow-up of the non-vaccinated subjects divided by the number of person-years of follow-up of the non-vaccinated cohort plus the pre-exposed person-years of the vaccinated cohort. As the mean follow-up period was longer for non-exposed versus exposed subjects, the numbers of cases for the 2-year analyses were calculated as N = events × 24 / follow-up (in months).

For GBS, a risk period of 42 days following each vaccination was considered for the main analysis. Given that 18% of the vaccinated cohort received 1 dose, 18% 2 doses, and 64% 3 doses, this gave a mean follow-up of 103 days per vaccinated individual. Among the non-exposed (non-vaccinated plus pre-vaccination) population, 21 GBS cases occurred (estimated 15.7 cases among non-vaccinated) during 30.2 months follow-up. The following calculation was used to estimate the number of GBS cases during the same time period: cases = 15.7 × 3.39 / 30.2 = 1.76.

**References**

1. Agence Nationale de Sécurité du Médicament et des produits de santé (ANSM). Vaccination contre les infections à HPV et risque de maladies auto-immunes : une étude Cnamts/ANSM rassurante - Point d'information. <http://ansm.sante.fr/S-informer/Points-d-information-Points-d-information/Vaccination-contre-les-infections-a-HPV-et-risque-de-maladies-auto-immunes-une-etude-Cnamts-ANSM-rassurante-Point-d-information> (accessed 26 August 2016).

2. Collin C, Miranda S, Zureik M, Dray-Spira R. HPV vaccines and the risk of thyroiditis in girls. Complementary analyses of the French cohort based on data from SNIIRAM. 2018.

3. Miranda S, Chaignot C, Collin C, Dray-Spira R, Weill A, Zureik M. Human papillomavirus vaccination and risk of autoimmune diseases: A large cohort study of over 2 million young girls in France. *Vaccine* 2017; **35**: 4761-4768.
