# Supplementary material for: Meta‐analysis of the risk of autoimmune thyroiditis, Guillain‐Barré syndrome, and inflammatory bowel disease following vaccination with AS04‐adjuvanted human papillomavirus 16/18 vaccine
Source: Pharmacoepidemiol Drug Saf. 2020 Jun 24;29(9):1159–67. doi: 10.1002/pds.5063 (PMC7539912; doi:10.1002/pds.5063)
Supplement: Supplementary file 1 — Data S1. Supporting Information. [file PDS-29-1159-s001.zip › PDS_5063_pds-19-0290-File007.docx]

**Supporting Information F.**

The continuity correction factor in the exposed arm, C_e_, was computed as C_e_ = 0.5 N_e_/N_T_ where N_e_ is the number of subjects in the exposed arm and N_T_ is the total number of subjects. The continuity correction factor in the non-exposed arm, C_ne_, was similarly computed by substituting N_e_ by N_ne_, the total number of subjects in the non-exposed arm. Both fixed- and random-effect models were used. The inverse-weighted variance method was used for the fixed-effect model. Heterogeneity among studies was tested using the Cochran *Q* test, the degree of heterogeneity by the *I²* index.^1^ For the random-effect model, a component of inter-study variance was added in the overall variance.

**Reference**

1. Huedo-Medina TB, Sanchez-Meca J, Marin-Martinez F, Botella J. Assessing heterogeneity in meta-analysis: *Q* statistic or *I^2^* index? *Psychol Methods* 2006; **11**: 193-206.
