# Supplementary material for: Meta‐analysis of the risk of autoimmune thyroiditis, Guillain‐Barré syndrome, and inflammatory bowel disease following vaccination with AS04‐adjuvanted human papillomavirus 16/18 vaccine
Source: Pharmacoepidemiol Drug Saf. 2020 Jun 24;29(9):1159–67. doi: 10.1002/pds.5063 (PMC7539912; doi:10.1002/pds.5063)
Supplement: Supplementary file 1 — Data S1. Supporting Information. [file PDS-29-1159-s001.zip › PDS_5063_pds-19-0290-File009.docx]

**Supporting information H.** Risk of GBS during ≤2 years following the first dose of AS04-HPV-16/18. CI, confidence interval; GBS, Guillain–Barré syndrome; OR, odds ratio; Randomized Controlled Trials; UK, United Kingdom ^1-18^ ^19,20^ ^21^ ^22,23^ ^24,25^


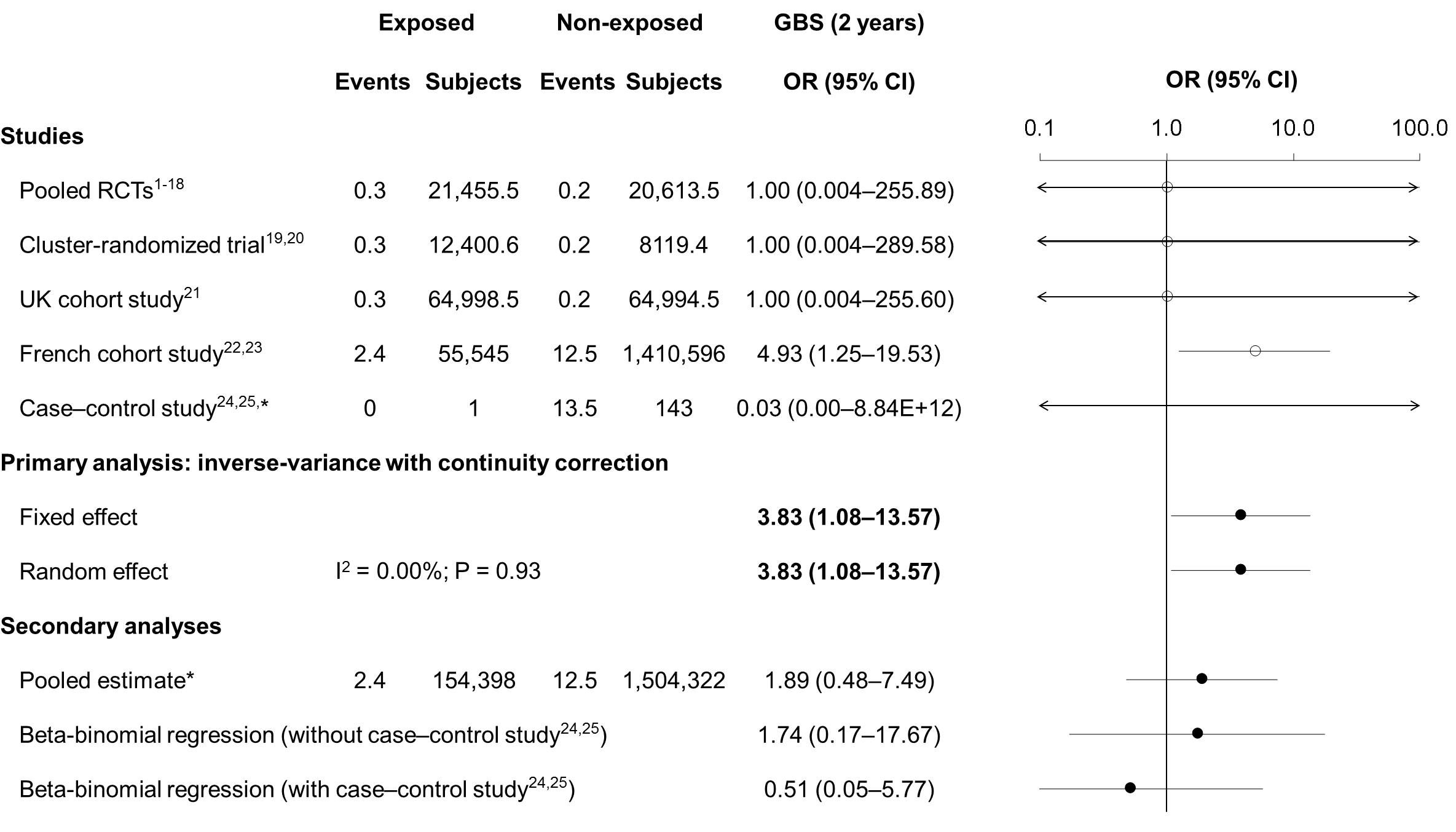


*The case–control study was not included in the pooled estimate.

**References**

1. Harper DM, Franco EL, Wheeler C, Ferris DG, Jenkins D, Schuind A, Zahaf T, Innis B, Naud P, De Carvalho NS, Roteli-Martins CM, Teixeira J, Blatter MM, Korn AP, Quint W, Dubin G, GlaxoSmithKline HPV Vaccine Study Group. Efficacy of a bivalent L1 virus-like particle vaccine in prevention of infection with human papillomavirus types 16 and 18 in young women: a randomised controlled trial. *Lancet* 2004; **364**: 1757-1765.

2. Harper DM, Franco EL, Wheeler CM, Moscicki AB, Romanowski B, Roteli-Martins CM, Jenkins D, Schuind A, Costa Clemens SA, Dubin G, HPV Vaccine Study group. Sustained efficacy up to 4.5 years of a bivalent L1 virus-like particle vaccine against human papillomavirus types 16 and 18: follow-up from a randomised control trial. *Lancet* 2006; **367**: 1247-1255.

3. Paavonen J, Naud P, Salmeron J, Wheeler CM, Chow SN, Apter D, Kitchener H, Castellsague X, Teixeira JC, Skinner SR, Hedrick J, Jaisamrarn U, Limson G, Garland S, Szarewski A, Romanowski B, Aoki FY, Schwarz TF, Poppe WA, Bosch FX, Jenkins D, Hardt K, Zahaf T, Descamps D, Struyf F, Lehtinen M, Dubin G, HPV PATRICIA Study Group. Efficacy of human papillomavirus (HPV)-16/18 AS04-adjuvanted vaccine against cervical infection and precancer caused by oncogenic HPV types (PATRICIA): final analysis of a double-blind, randomised study in young women. *Lancet* 2009; **374**: 301-314.

4. Herrero R, Wacholder S, Rodriguez AC, Solomon D, Gonzalez P, Kreimer AR, Porras C, Schussler J, Jimenez S, Sherman ME, Quint W, Schiller JT, Lowy DR, Schiffman M, Hildesheim A, Costa Rica Vaccine Trial Group. Prevention of persistent human papillomavirus infection by an HPV16/18 vaccine: a community-based randomized clinical trial in Guanacaste, Costa Rica. *Cancer Discov* 2011; **1**: 408-419.

5. Medina DM, Valencia A, de Velasquez A, Huang LM, Prymula R, Garcia-Sicilia J, Rombo L, David MP, Descamps D, Hardt K, Dubin G, HPV Study Group. Safety and immunogenicity of the HPV-16/18 AS04-adjuvanted vaccine: a randomized, controlled trial in adolescent girls. *J Adolesc Health* 2010; **46**: 414-421.

6. Skinner SR, Szarewski A, Romanowski B, Garland SM, Lazcano-Ponce E, Salmeron J, Del Rosario-Raymundo MR, Verheijen RH, Quek SC, da Silva DP, Kitchener H, Fong KL, Bouchard C, Money DM, Ilancheran A, Cruickshank ME, Levin MJ, Chatterjee A, Stapleton JT, Martens M, Quint W, David MP, Meric D, Hardt K, Descamps D, Geeraerts B, Struyf F, Dubin G, VIVIANE Study Group. Efficacy, safety, and immunogenicity of the human papillomavirus 16/18 AS04-adjuvanted vaccine in women older than 25 years: 4-year interim follow-up of the phase 3, double-blind, randomised controlled VIVIANE study. *Lancet* 2014; **384**: 2213-2227.

7. Denny L, Hendricks B, Gordon C, Thomas F, Hezareh M, Dobbelaere K, Durand C, Herve C, Descamps D. Safety and immunogenicity of the HPV-16/18 AS04-adjuvanted vaccine in HIV-positive women in South Africa: a partially-blind randomised placebo-controlled study. *Vaccine* 2013; **31**: 5745-5753.

8. Sow PS, Watson-Jones D, Kiviat N, Changalucha J, Mbaye KD, Brown J, Bousso K, Kavishe B, Andreasen A, Toure M, Kapiga S, Mayaud P, Hayes R, Lebacq M, Herazeh M, Thomas F, Descamps D. Safety and immunogenicity of human papillomavirus-16/18 AS04-adjuvanted vaccine: a randomized trial in 10-25-year-old HIV-seronegative African girls and young women. *J Infect Dis* 2013; **207**: 1753-1763.

9. Leroux-Roels G, Haelterman E, Maes C, Levy J, De Boever F, Licini L, David MP, Dobbelaere K, Descamps D. Randomized trial of the immunogenicity and safety of the Hepatitis B vaccine given in an accelerated schedule coadministered with the human papillomavirus type 16/18 AS04-adjuvanted cervical cancer vaccine. *Clin Vaccine Immunol* 2011; **18**: 1510-1518.

10. Pedersen C, Breindahl M, Aggarwal N, Berglund J, Oroszlan G, Silfverdal SA, Szuts P, O'Mahony M, David MP, Dobbelaere K, Dubin G, Descamps D. Randomized trial: immunogenicity and safety of coadministered human papillomavirus-16/18 AS04-adjuvanted vaccine and combined hepatitis A and B vaccine in girls. *J Adolesc Health* 2012; **50**: 38-46.

11. Schmeink CE, Bekkers RL, Josefsson A, Richardus JH, Berndtsson Blom K, David MP, Dobbelaere K, Descamps D. Co-administration of human papillomavirus-16/18 AS04-adjuvanted vaccine with hepatitis B vaccine: randomized study in healthy girls. *Vaccine* 2011; **29**: 9276-9283.

12. Bhatla N, Suri V, Basu P, Shastri S, Datta SK, Bi D, Descamps DJ, Bock HL, Indian HPV Vaccine Study Group. Immunogenicity and safety of human papillomavirus-16/18 AS04-adjuvanted cervical cancer vaccine in healthy Indian women. *J Obstet Gynaecol Res* 2010; **36**: 123-132.

13. Konno R, Tamura S, Dobbelaere K, Yoshikawa H. Efficacy of human papillomavirus type 16/18 AS04-adjuvanted vaccine in Japanese women aged 20 to 25 years: final analysis of a phase 2 double-blind, randomized controlled trial. *Int J Gynecol Cancer* 2010; **20**: 847-855.

14. Kim YJ, Kim KT, Kim JH, Cha SD, Kim JW, Bae DS, Nam JH, Ahn WS, Choi HS, Ng T, Bi D, Ok JJ, Descamps D, Bock HL. Vaccination with a human papillomavirus (HPV)-16/18 AS04-adjuvanted cervical cancer vaccine in Korean girls aged 10-14 years. *J Korean Med Sci* 2010; **25**: 1197-1204.

15. Ngan HY, Cheung AN, Tam KF, Chan KK, Tang HW, Bi D, Descamps D, Bock HL. Human papillomavirus-16/18 AS04-adjuvanted cervical cancer vaccine: immunogenicity and safety in healthy Chinese women from Hong Kong. *Hong Kong Med J* 2010; **16**: 171-179.

16. Lim BK, Ng KY, Omar J, Omar SZ, Gunapalaiah B, Teoh YL, Bock HL, Bi D. Immunogenicity and Safety of the AS04-adjuvanted Human Papillomavirus-16/18 Cervical Cancer Vaccine in Malaysian Women Aged 18-35 years: A Randomized Controlled Trial. *Med J Malaysia* 2014; **69**: 2-8.

17. Kim SC, Song YS, Kim YT, Kim YT, Ryu KS, Gunapalaiah B, Bi D, Bock HL, Park JS. Human papillomavirus 16/18 AS04-adjuvanted cervical cancer vaccine: immunogenicity and safety in 15-25 years old healthy Korean women. *J Gynecol Oncol* 2011; **22**: 67-75.

18. Zhu F, Li J, Hu Y, Zhang X, Yang X, Zhao H, Wang J, Yang J, Xia G, Dai Q, Tang H, Suryakiran P, Datta SK, Descamps D, Bi D, Struyf F. Immunogenicity and safety of the HPV-16/18 AS04-adjuvanted vaccine in healthy Chinese girls and women aged 9 to 45 years. *Hum Vaccin Immunother* 2014; **10**: 1795-1806.

19. Lehtinen M, Apter D, Baussano I, Eriksson T, Natunen K, Paavonen J, Vanska S, Bi D, David MP, Datta S, Struyf F, Jenkins D, Pukkala E, Garnett G, Dubin G. Characteristics of a cluster-randomized phase IV human papillomavirus vaccination effectiveness trial. *Vaccine* 2015; **33**: 1284-1290.

20. GlaxoSmithKline Biologicals. An observational cohort study to assess the risk of autoimmune diseases in adolescent and young adult women aged 9 to 25 years exposed to Cervarix® in the United Kingdom. Study number 116239 (EPI-HPV-040 VS UK). <https://gsk.sylogent.com/files/116239-Clinical-Study-Result-Summary.pdf> (accessed 25 October 2016).

21. Willame C, Rosillon D, Zima J, Angelo MG, Stuurman AL, Vroling H, Boggon R, Bunge EM, Pladevall-Vila M, Baril L. Risk of new onset autoimmune disease in 9- to 25-year-old women exposed to human papillomavirus-16/18 AS04-adjuvanted vaccine in the United Kingdom. *Hum Vaccin Immunother* 2016; **12**: 2862-2871.

22. Agence Nationale de Sécurité du Médicament et des produits de santé (ANSM). Vaccination contre les infections à HPV et risque de maladies auto-immunes : une étude Cnamts/ANSM rassurante - Point d'information. <http://ansm.sante.fr/S-informer/Points-d-information-Points-d-information/Vaccination-contre-les-infections-a-HPV-et-risque-de-maladies-auto-immunes-une-etude-Cnamts-ANSM-rassurante-Point-d-information> (accessed 26 August 2016).

23. Miranda S, Chaignot C, Collin C, Dray-Spira R, Weill A, Zureik M. Human papillomavirus vaccination and risk of autoimmune diseases: A large cohort study of over 2 million young girls in France. *Vaccine* 2017; **35**: 4761-4768.

24. Grimaldi-Bensouda L, Rossignol M, Kone-Paut I, Krivitzky A, Lebrun-Frenay C, Clet J, Brassat D, Papeix C, Nicolino M, Benhamou PY, Fain O, Costedoat-Chalumeau N, Courcoux MF, Viallard JF, Godeau B, Papo T, Vermersch P, Bourgault-Villada I, Breart G, Abenhaim L, PGRx-AD Study Group. Risk of autoimmune diseases and human papilloma virus (HPV) vaccines: Six years of case-referent surveillance. *J Autoimmun* 2017; **79**: 84-90.

25. Grimaldi-Bensouda L, Aubrun E, Abenhaim L. ANALYSIS OF CERVARIX® & AUTOIMMUNE DISORDERS USING THE PGRx INFORMATION SYSTEM. Data on file. 2015.
